# Mitochondrial Dysfunction Inhibits Hypoxia-Induced HIF-1α Stabilization and Expression of Its Downstream Targets

**DOI:** 10.3389/fonc.2020.00770

**Published:** 2020-05-19

**Authors:** Marike W. van Gisbergen, Kelly Offermans, An M. Voets, Natasja G. Lieuwes, Rianne Biemans, Roland F. Hoffmann, Ludwig J. Dubois, Philippe Lambin

**Affiliations:** ^1^The M-Lab, Department of Precision Medicine, GROW—School for Oncology and Developmental Biology, Maastricht University, Maastricht, Netherlands; ^2^Department of Clinical Genomics, GROW—School for Oncology and Developmental Biology, Maastricht University Medical Centre, Maastricht, Netherlands; ^3^Department of Pathology and Medical Biology, University of Groningen, University Medical Center Groningen, Groningen, Netherlands

**Keywords:** mtDNA, mitochondria, OXPHOS, CAIX, HIF-1α, Metformin

## Abstract

mtDNA variations often result in bioenergetic dysfunction inducing a metabolic switch toward glycolysis resulting in an unbalanced pH homeostasis. In hypoxic cells, expression of the tumor-associated carbonic anhydrase IX (CAIX) is enhanced to maintain cellular pH homeostasis. We hypothesized that cells with a dysfunctional oxidative phosphorylation machinery display elevated CAIX expression levels. Increased glycolysis was observed for cytoplasmic 143B mutant hybrid (m.3243A>G, >94.5%) cells (*p* < 0.05) and 143B mitochondrial DNA (mtDNA) depleted cells (*p* < 0.05). Upon hypoxia (0.2%, 16 h), genetic or pharmacological oxidative phosphorylation (OXPHOS) inhibition resulted in decreased CAIX (*p* < 0.05), vascular endothelial growth factor (VEGF) and hypoxia-inducible factor 1-alpha (HIF-1α) expression levels. Reactive oxygen species (ROS) and prolyl-hydroxylase 2 (PHD2) levels could not explain these observations. *In vivo*, tumor take (>500 mm^3^) took longer for mutant hybrid xenografts, but growth rates were comparable with control tumors upon establishment. Previously, it has been shown that HIF-1α is responsible for tumor establishment. In agreement, we found that HIF-1α expression levels and the pimonidazole-positive hypoxic fraction were reduced for the mutant hybrid xenografts. Our results demonstrate that OXPHOS dysfunction leads to a decreased HIF-1α stabilization and subsequently to a reduced expression of its downstream targets and hypoxic fraction *in vivo*. In contrast, hypoxia-inducible factor 2-alpha (HIF-2α) expression levels in these xenografts were enhanced. Inhibition of mitochondrial function is therefore an interesting approach to increase therapeutic efficacy in hypoxic tumors.

## Introduction

As the tumor microenvironment is a heterogeneous and dynamic entity, cells within a tumor have different gene expression profiles, metabolism and oxygen supply. The lack of nutrient and oxygen supply is associated with two main characteristics of a malignancy, namely bioenergetics and angiogenesis (1). The adaptation of cancer cells to hypoxia, the low oxygen regions within a tumor and its metabolic consequences, is critical for tumor progression. Tumors cells in a hypoxic tumor microenvironment have often the capability to alter their metabolism and favor metabolic pathways, which are less dependent on oxygen, to meet their energy demand. Hypoxic tumor cells often produce large amount of acids due to their increase in glycolysis and have a decreased extracellular pH (2–4). One important family of proteins involved in tumor pH maintenance are carbonic anhydrases (CA) (5). The membrane bound zinc-metallo-enzyme CAIX is capable of re-hydrating CO_2_ into bicarbonate (${\text{HCO}}_{3}^{-}$) and a proton (H^+^) upon passive CO_2_ diffusion out of the cell. ${\text{HCO}}_{3}^{-}$ will subsequently re-enter the cell in order to neutralize the intracellular pH. The remaining proton acidifies the extracellular environment contributing to the worse prognosis in cancer patients (6, 7). High CAIX expression results in a higher risk of locoregional failure, disease progression and metastases development in cancer patients (8). In hypoxic tumors, CAIX expression is found to be upregulated through transcriptional activation upon interaction of hypoxia inducible factor-1α (HIF-1α) with the hypoxia response element (HRE) identified in its promotor region (9). In addition, the transport of lactate into the microenvironment via monocarboxylate transporter 4 (MCT-4) leads to a further increase in extracellular acidification. The role of dysfunctional mitochondria in these mechanisms is of interest, as it has been suggested that dysfunction of the oxidative phosphorylation machinery contributes to tumoral metabolic reprogramming (10, 11).

During hypoxic stress, the low efficiency glycolytic pathway is further upregulated and is mainly driven by HIF-1α. The high rate of glycolysis, which persists in tumor cells even under re-oxygenation, is associated with an increase in glucose uptake and lactate release. Lactic acidosis is a symptom often observed in patients with mitochondrial diseases such as the myoclonic epilepsy with ragged-red fibers (MERFF) syndrome (12). A cybrid model harboring a m.8344A>G mutation, encoding for ND5 subunit of Complex I (CI), corresponding to the MERFF phenotype in patients resulting in CI dysfunction, displayed reduced levels of carbonic anhydrase VIII (CAVIII) (13). Furthermore, it has been suggested that CAVIII regulates cellular stress responses and could also play a role in metabolism. CAVIII knockdown cells were found to have a decreases glycolytic activity and increased cell death under reduced glucose concentrations *in vitro* (14). These data suggest that mitochondrial dysfunction leading to decreased carbonic anhydrase expression levels could also be observed for other CA forms. Here, we investigated if the membrane-bound carbonic anhydrase IX (CAIX) was upregulated in cells with a mitochondrial dysfunction in order preserve to cellular functionality.

## Materials and Methods

### *In vitro* Experiments

#### mtDNA Depleted Cells

The parental and mtDNA depleted (rho-zero, ρ^0^) 143B osteosarcoma cell lines were kindly provided by Dr. Valeria Tiranti (Milan, Italy). Both cell lines were cultured in Gibco's Dulbecco's modified Eagle's medium (DMEM) with 10% fetal bovine serum (FBS; Sigma-Aldrich). Culture medium of the mtDNA depleted cells was supplemented with 150 μg/ml uridine (Acros Organics) and 100 μg/ml bromodeoxyuridine (Sigma-Aldrich). The parental and mtDNA depleted A549 (alveolar type-II carcinoma) cell lines were cultured in DMEM supplemented with 25% FBS, vitamins, amino acids (Sigma-Aldrich) and uridine (50 μg/ml; Acros Organics). mtDNA depletion was sustained by culturing cells in medium supplemented with ethidium bromide (50 ng/ml; Sigma-Aldrich). Fluorescent confocal imaging and quantitative PCR were used to confirm mtDNA depletion at several time points during the experiments. For confocal imaging, cells were stained (45 min) with MitoTracker® Deep-Red FM and Picogreen (Invitrogen) in D-PBS (GIBCO) according to the manufacturer's protocol. Subsequently, cells were washed twice with D-PBS and visualized using a Leica AOBS confocal microscope. Overlays were generated using Image J. Ratios of nDNA (B2M) and mtDNA (D-Loop) were obtained by quantitative PCR in order to determine the mtDNA copy numbers. DNA was isolated using the gentra puregene kit (Qiagen). Quantitative PCR was performed on the 7900HT Fast Real-Time PCR System (Applied Biosystems). Gene abundances were detected using the SensiMix SYBR® HiRox kit (Bioline Reagents). The cycling conditions were: 50°C (2 min), 95°C (10 s), 40 cycles at 95°C (15 s) and 60°C (1 min). Primer sequences for B2M and D-Loop can be found in Table S1.

#### Cytoplasmic Hybrids (Cybrids) and Quantification of Mutant mtDNA Percentage

Cytoplasmic hybrids cell lines (cybrids) were generated as previously described (15, 16). In short, cybrids were generated by fusing the mtDNA from patient fibroblasts with mtDNA depleted 143B cells. Two control cybrid cell lines were generated using fibroblast mtDNA from healthy volunteers (kindly provided by Dr. Hubertus Smeets, Maastricht, the Netherlands), while 2 mutant cybrid cell lines were created using fibroblasts harboring the m.3243A>G mutation encoding for mitochondrial tRNA-Leu(UUR) (*MT-TL1)* and responsible for the “mitochondrial encephalomyopathy, lactic acidosis, and stroke-like episodes” (MELAS) syndrome (17). Fibroblasts originated from two independent patients harboring the m.3243A>G mutation, resulted in two independent mutant cybrid cell lines, named MELAS 1 and MELAS 2. All cell lines were cultured in DMEM supplemented with 5% dialyzed serum (Invitrogen). To determine the percentage of mtDNA harboring the MELAS m.3243A>G mutation, the coding mtDNA sequence was amplified using primer sequences found in Table S1. The PCR amplification consisted of an initial denaturation at 94°C (5 min), followed by 32 cycles at 92°C (1 min), 53°C (1 min), 72°C (45 s) and a final elongation at 72°C (7 min). A FAM-labeled forward primer was added to the first PCR product, which was subjected to an additional PCR cycle. Gel electrophoresis (2% agarose) confirmed size of the PCR products. Fragments were excised from the gel and digested (2.5 h at 37°C) using a mix of sterile water, digestion buffer and the restriction enzyme Hae III. Digestion products were purified using the QiaQuick PCR Purification kit (Qiagen) and analyzed with ABI 3730 using a G5 filter. The primer sequences for fragment analysis can be found in Table S1.

#### Metabolic Profiling

Metabolic profiles were generated using the Seahorse XF96 extracellular Flux analyzer (Agilent) according to manufacturer's guidelines (18). Cells were seeded at an optimized cell density of 30.000 cells/well. Basal respiration rates were determined as described previously (19). The glycolysis stress test was performed by sequential addition of 10 mM glucose, optimized oligomycin concentration (2.5 μM) and 0.1 M 2-deoxyglucose (2-DG) (Sigma-Aldrich).

#### OXPHOS Inhibition

Five hundred thousand cells of each cell line were seeded in 6 cm centimeter dishes. Cells were exposed to either normoxia (20% O_2_) or hypoxia (0.2% O_2_) for 16 h simultaneously with incubation of vehicle (0.02% DMSO), 5 mM metformin (Sigma-Aldrich) or 1 μM rotenone (Sigma-aldrich). After 16 h, cells were immediately put on ice and were washed with PBS prior to protein or RNA isolation.

#### DNA Isolation, RNA Isolation and Quantitative PCR Analysis

mRNA was extracted using the NucleoSpin RNA II kit (Bioke) and reverse transcribed using the iScript cDNA Synthesis Kit (BioRad). All procedures were according to the manufacturers' instructions. Quantitative RT-PCR was performed in the ABI 7700 (Applied Biosystems) or 7900HT Fast Real-Time PCR. Gene abundances were detected with SYBR® Green (Eurogentec). mRNA expression was either normalized to 18S or Actin. Primer sequences can be found in Table S1.

#### SDS-PAGE and Western Blotting

Cells were lysed and processed as described previously (20, 21). Antibodies used were M75 (1:40) (kindly provided by Silvia Pastorekova, Institute of Virology, Slovak Academy of Science, Bratislava, Slovak Republic) against CAIX, anti-HIF-1α (1:250, BD transduction), anti-HIF-2α (1:1.000, NB100-122, Novus biologicals) and anti-β-actin (1:200.000, MP biomedicals). Proteins were visualized by the horseradish peroxidase method (anti-mouse or anti-rabbit, both 1:2.000, Cell Signaling) by using ECL prime western blotting detection reagent (Amersham Corp) or ECL supersignal west pico (Thermo Fisher). Complete Western Blots can be found in Data Sheet 1.

#### Flow Cytometry

Formation of reactive oxygen species (ROS) was detected 16 h after hypoxia or normoxia exposure. Cells were incubated with 20 μM dihydrorhodamine-123 during the last hour of exposure. ROS levels were determined in the propidium iodide (Sigma-Aldrich) negative population by flow cytometry (BD FACS Canto II).

#### Hypoxia Tolerance

Cells were seeded in 6 cm dishes, allowed to attach under normoxic conditions overnight and exposed to hypoxic conditions (0.2% O_2_) for 24, 48, or 72 h. After hypoxic exposure, medium was replaced and cells were allowed to form colonies under normoxia for 7 days. Colonies were quantified after staining and fixation with 0.4% methylene blue in 70% ethanol. A colony was defined as > 50 cells.

### *In vivo* Experiments

#### *In vivo* Models

Control and mutant cybrid cells (1.5^*^10^6^) were resuspended in Basement Membrane Matrix (Matrigel™ BD Biosciences) and injected subcutaneously into the lateral flank of adult NMRI nu/nu mice (28–30 g). Tumor growth was monitored until reaching a volume of 1.2 cm^3^.

#### Immunohistochemisty

Frozen xenograft tumors were sectioned (5 μm) and stained for hypoxia (pimonidazole) and CAIX. Sections were fixed using cold acetone, rehydrated in TBS with 0.2% Tween-20 (TBS-T) and pre-incubated with 1% normal goat serum (NGS) before exposing them to the primary antibodies: FITC-conjugated IgG1 mouse monocolonal anti-pimonidazole (1:150, clone 4.3.11.3, HP1-Plus Kit, Bio-connect) and rabbit polyclonal anti-CAIX (1:1.000, Novus biologicals). After washing with TBS-T, incubation with the secondary antibody goat anti-rabbit Alexa 594 (1:500) (Invitrogen) was performed. Sections were mounted using fluorescent mounting medium (DakoCytomation) and digitally scanned using an Olympus BX51WI fluorescence microscope with a Hamamatsu EM-CCD C9100 digital camera, a motorized stage (Ludl Mac 2000) and a 10x objective. Micromanager 1.4 software was used for automated image acquisition. Image stitching was performed by using ImageJ software.

#### Statistics

All statistical analyses were performed with GraphPad Prism (GraphPad Software, version 5.03, 2009, California, USA). Student's *t*– test was used to determine the statistical significance of differences between two independent groups of variables. A *p*-value < 0.05 was considered significant.

## Results

**Figure 1:**
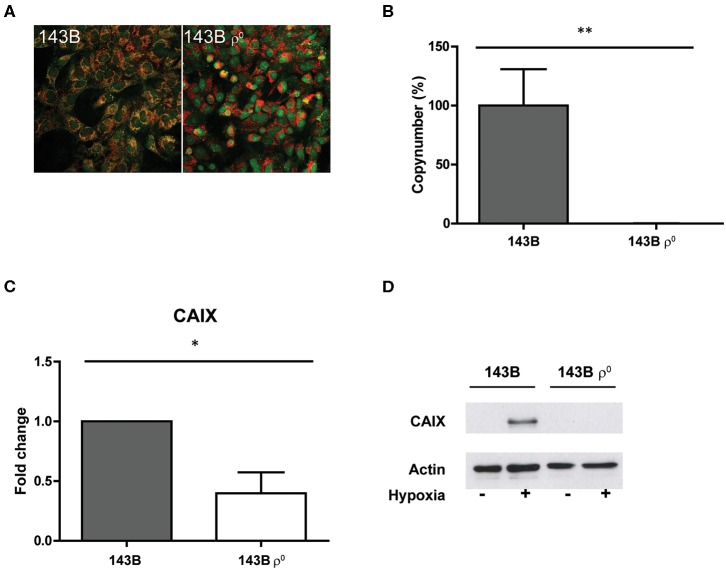
Generation and characterization of a mtDNA depleted cell line. **(A)** Merged images of dsDNA staining (green) and mitochondria (red). **(B)** Percentage mtDNA copy number. **(C)** CAIX mRNA expression upon hypoxia. **(D)** Representative Western blot of CAIX protein expression upon normoxia (–) or hypoxia (+). Data represent the mean ± SEM of ≥3 biological repeats. *p < 0.05, **p < 0.01.

In order to investigate if extreme changes in oxidative phosphorylation (OXPHOS) capacity would lead to a change in CAIX expression, we used 143B and A549 mitochondrial DNA (mtDNA) depleted cancer cell lines (ρ^0^ cells) (Figure 1; Figure S1). Both cell lines showed a substantial decrease of mtDNA (Figure 1A, Figure S1A), which was confirmed by the significant (*p* < 0.01) decrease in mtDNA copy number (Figure 1B and Figure S1B). Hypoxia-induced CAIX expression was decreased (*p* < 0.05) in the cells depleted from their mtDNA, at both mRNA (Figure 1C, Figure S1C) and protein levels (Figure 1D, Figures S1D, S2) as compared to their parental counterparts.

**Figure 2:**
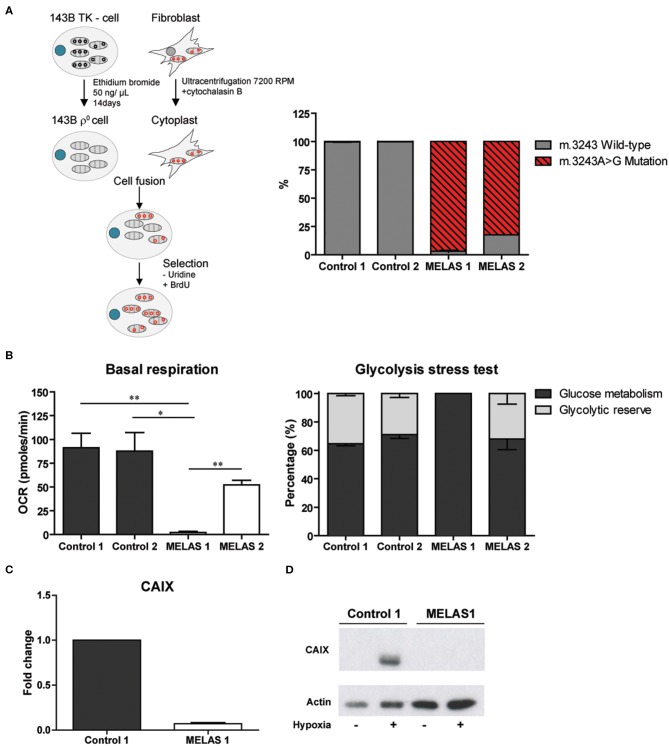
Generation and characterization of the cybrid model and influence on CAIX expression. **(A)** Procedure to generate MELAS and control cybrid cell lines (left) and percentage m.3243A>G mutated mtDNA in these cybrid lines (right). **(B)** Metabolic profiling of the cybrid lines, showing basal respiration (left) and glycolysis stress test results (right). **(C)** CAIX mRNA expression upon hypoxia. **(D)** Representative Western blot of CAIX protein expression upon normoxia (–) or hypoxia (+). Data represents mean + SEM of ≥3 independent biological repeats. *p < 0.05, **p < 0.01.

Our observations were extended to a second model, in which mtDNA is not abolished but harbors a point mutation, the so-called cytoplasmic hybrid or cybrid model. For this purpose, the mtDNA depleted 143B osteosarcoma cell line was fused with the cytoplasts of a fibroblast cell line from a MELAS patient carrying a m.3243A>G mutation. The generated mutant cybrid polyclonal cell line MELAS 1 had an average mutation load of 96.80%, while the control cybrid clones contained 99.85 (control 1) and 99.65% (control 2) of the wild-type sequence. The independently obtained MELAS 2 cells showed a mutation load of 82.33% (Figure 2A). To further characterize the cybrid models, metabolic activity was investigated by assessing mitochondrial respiration and glycolytic capacity. Basal respiration of the MELAS 1 cells was significantly (*p* < 0.05) reduced compared to the controls (Figure 2B and Figure S3), a functional consequence that also was observed for not only the m.3243A>G mutation but also for cybrids having a frameshift (m.3571insC/MT-ND1) mutation (22). Where MELAS 1 cells were completely dependent on glycolysis, since no glycolytic reserve capacity could be observed (Figure 2B), MELAS 2 did display a glycolytic reserve capacity with comparable levels to the control cell lines, suggesting that the OXPHOS was still functional in these cells. Baseline respiration and glycolysis was similar for both control cybrid lines. Upon hypoxia, CAIX mRNA and protein expression were reduced in MELAS 1 cells as compared to the control cybrid cells (Figures 2C,D).

**Figure 3:**
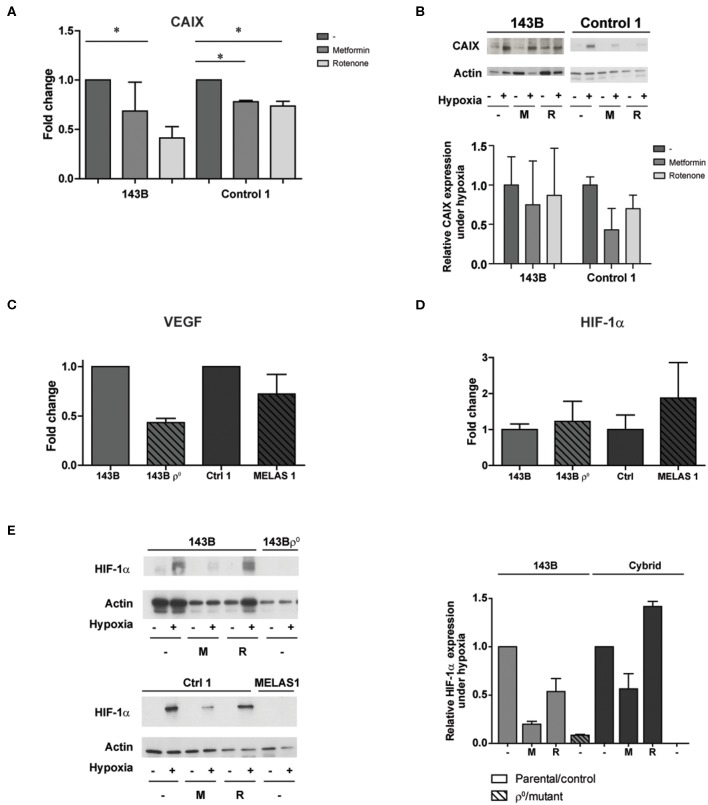
The influence of pharmacological CI inhibition on CAIX and other HIF-1α targets upon low oxygen levels. **(A)** CAIX mRNA expression upon hypoxia with or without Metformin (5 mM) or Rotenone (1 μM). **(B)** Representative Western blot and quantification of CAIX protein expression upon normoxia (–) or hypoxia (+) with or without Metformin (M, 5 mM) or Rotenone (R, 1μM). **(C)** VEGF and **(D)** HIF-1α mRNA expression under hypoxia. **(E)** Representative Western blot and quantification of HIF-1α protein expression upon normoxia (–) or hypoxia (+), combined with exposure of vehicle (–), metformin (M) or rotenone (R). Data represents the mean of ≥2 independent biological repeats + SEM. *p < 0.05.

For MELAS 2 mutants, harboring a lower mutation percentage, this effect was rescued (Figure S4) and therefore these mutant cells were excluded from further experiments. A MELAS mutation leads to a reduction in activity of Complex I–IV of the oxidative phosphorylation chain (17). In order to investigate if the observed effects from the genetic approach could be mimicked by pharmacological inhibition, wild-type cells were exposed to either metformin or rotenone, both complex I (CI) inhibitors. Pharmacological CI inhibition resulted in reduced CAIX mRNA and protein expression upon hypoxia exposure in the 143B parental cells (rotenone *p* < 0.05) and the control cybrid line (metformin and rotenone *p* < 0.05) (Figures 3A,B). CAIX expression was not reduced for the A549 cells (Figure S1E). Additionally, mRNA expression levels for carbonic anhydrase XII (CAXII), another hypoxia inducible carbonic anhydrase (23), were upregulated for 143B mtDNA depleted cells and cells harboring a m.3243A>G mutation under hypoxia, but not for cells exposed to the CI inhibitor rotenone (Figure S5). Vascular endothelial growth factor (VEGF) mRNA expression, another HIF-1α downstream target next to CAIX, was also decreased (Figure 3C). HIF-1α protein expression upon hypoxia was reduced for the parental lines and the control cybrid line after exposure to metformin. HIF-1α mRNA expression was not affected (Figure 3D), while HIF-1α protein expression was in general low for MELAS 1 mutant cells (Figure 3E).

**Figure 4:**
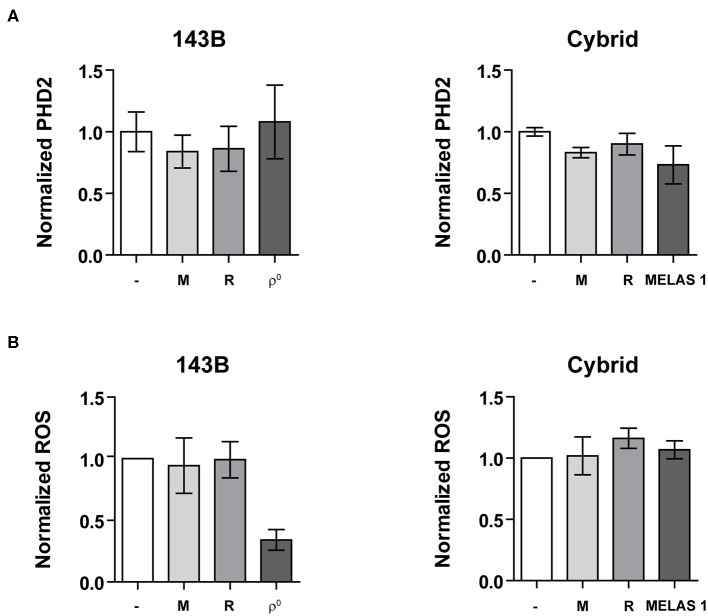
HIF-1α regulators. **(A)** Normalized PHD2 mRNA expression and **(B)** Normalized ROS production upon mtDNA depletion (left) or mtDNA mutation (right) under hypoxic conditions with or without OXPHOS CI inhibitors metformin (M) or rotenone (R). Data are normalized to either 143B (left) or ctrl 1 cybrid cell lines (right). Data represents the mean of ≥2 independent biological repeats ± SEM.

Prolyl-hydroxylase 2 (PHD2) is the main regulator of HIF-1α stabilization (24–26). Therefore, we investigated if changes in PHD2 expression could explain the observed reduction in HIF-1α stabilization and expression of its downstream targets. PHD2 mRNA expression was not changed upon genetic imposed mitochondrial dysfunction (Figure 4A). Similarly, mRNA expression of PHD1 and PHD3 did not alter (data not shown). Another major contributor to HIF-1α stabilization is the presence of ROS production (27, 28). Contradictory results have been reported on the influence of mtDNA variations on ROS production, which can be increased (17, 29) or decreased (30, 31). Hypoxia-induced ROS production was slightly decreased (*P* = 0.0636) for the mtDNA depleted as compared to the parental 143B cells, while no differences were observed between the cybrid cell lines (control vs. MELAS 1) (Figure 4B, Figure S6). Additionally, pharmacological inhibition was not able to change ROS production.

**Figure 5:**
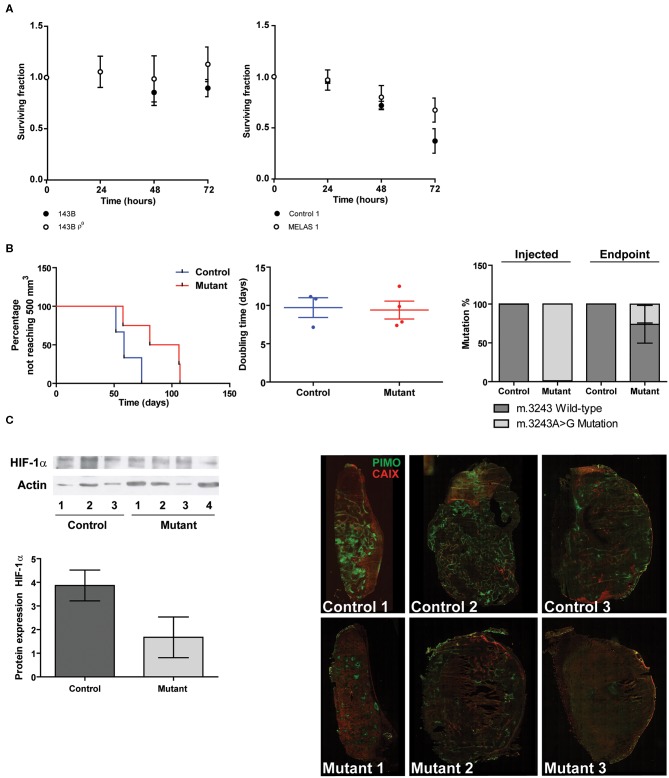
Functional consequences of mtDNA mutations in relation to hypoxia. **(A)** Hypoxia tolerance. Data represents the mean of ≥3 independent biological repeats ± SEM. **(B)** Percentage not reaching 500 mm^3^ (left panel), Doubling time (middle panel), and mutation percentage before injection and after tumor excision (right panel). **(C)** Representative Western blot and quantification of HIF-1α protein expression (left). Immunohistochemisty (right) of hypoxia assessed using pimonidazole (green) and CAIX (red).

In order to investigate the functional consequences of decreased CAIX expression, we assessed hypoxia tolerance. Survival under prolonged hypoxia for parental and mtDNA depleted 143B cells was not altered. On the other hand, the mutant cybrid models rescued the lower survival of the control cybrid line (Figure 5A). *In vivo*, MELAS cybrid xenografts needed longer to reach 500 mm^3^ compared to control cybrid tumors, however no differences were found in doubling time upon tumor establishment suggesting a delayed tumor take (*P* = 0.09, Figure 5B). Additionally, the mutation percentage for the mutant hybrid xenografts at sacrifice was decreased compared with the injected cells (Figure 5B). HIF-1α protein was reduced in the mutant hybrid xenografts compared with control tumors, however a large variation was observed (Figure 5C), while an increase in hypoxia-inducible factor-2α (HIF-2α) expression was observed for the mutant xenografts (Figure S7). Additionally, we observed that in the MELAS cybrids xenografts pimonidazole-positive hypoxic areas were reduced. However, no large differences upon CAIX staining could be observed (Figure 5C).

Alterations in overall mRNA translation are influenced upstream by the unfolded protein response (UPR), a mechanism influenced by hypoxia and energetic stress (32–34). Additionally, there is also a specific mitochondrial UPR response. One of the regulators influencing this mitochondrial UPR is NAD-dependent deacetylase sirtuin-3 (SIRT3) (35). SIRT3 is located in the mitochondria and is involved in various cellular mechanisms such as nutrient stress (36), fatty acid oxidation (37), AMPK activation (38), anti-oxidant mechanisms (39) and in the activation of a hypoxia-induced mitochondrial form of autophagy (mitophagy) (40). No major hypoxia-induced differences could be observed for SIRT3 mRNA expression levels (Figure S8), however MELAS1 showed a reduced SIRT3 expression upon hypoxia.

## Discussion

In the present study, the effect of OXPHOS inhibition using genetic models (mtDNA depleted cells and mtDNA mutated cytoplasmic hybrids) or pharmacological inhibitors (rotenone or metformin) on CAIX expression and HIF-1α stabilization was investigated. We hypothesized that a decrease of mitochondrial respiration through OXPHOS would lead to an increase in CAIX expression upon hypoxia exposure due to an induced metabolic shift. However, contradicting our hypothesis we observed that CAIX expression as well as HIF-1α levels were reduced in cells with extreme mitochondrial dysfunction, which seems to be heteroplasmy dependent, when exposed to low (≤0.2 O_2_) oxygen tension. We also observed a simultaneous increase of CAXII expression, which can be a compensation measure for CAIX down regulation (41). Since the tumor microenvironment is a heterogeneous and dynamic mechanism, cells within a tumor can have different gene expression profiles, metabolism and oxygen supply (42–44). The adaptation of cancer cells to for example hypoxia is critical for tumor progression and metastasis formation and is regulated by hypoxia-inducible factor-1 (HIF-1) (45–48). Under reduced oxygen conditions, normally HIF-1α is stabilized (2, 49).

Different studies have described an association between HIF-1 and mitochondrial function. Genetic modulation of mitochondrial function, both through mtDNA depletion, mutations and/or nDNA mutations, could potentially serve as an interesting proof of concept strategy. In our study, mtDNA variation and depletion indeed showed HIF-1α de-stabilization. This was confirmed in a recent proof of concept study in 2 different tumor backgrounds (HCT116 and 143B) having a nuclear-encoded *NDUFS3* knock-out, where HIF-1α stabilization was abolished, but that tumors were able to re-adapt to the hypoxia response (50). These adaptations to hypoxia are supported by the observed increase in HIF-2 expression in the cybrid mutant xenografts, which also has been evidenced from our recently published work (21).

Next to genetic alterations to induce impairment of OXPHOS function and subsequently cause a reduced HIF-1 stabilization, pharmacological inhibitors are also able to cause this effect. For instance it has been shown that BAY 87-2243, a potent inhibitor of HIF-1α, reduced tumor growth, potentially through targeting mitochondrial complex I (CI) (51, 52). Similarly, the CI inhibitor Kalkitoxin reduced tumor cell proliferation under hypoxia, but was also capable of reducing HIF1 stabilization (53). The CI inhibitor AG311 also reduced HIF-1α stabilization and in combination with an inhibitor of the pyruvate dehydrogenase kinase, a key regulatory enzyme of oxidative metabolism, resulted in a reduced tumor growth (54). The potentially most clinical interesting CI inhibitor, metformin, also used in our study, has already shown its ability to decrease HIF-1α stabilization in various tumor backgrounds (55, 56) and therefore contributes to a slower tumor growth, potentially related to the suppression of VEGF (56–58). However, a tumor-dependent effect might be present as we did not observe reduced levels of CAIX and VEGF upon metformin exposure in all tumor cell types. This is in line with findings of Khan et al., that indicate that biguanide-induced mitochondrial CI dysfunction stabilizes HIF-1α through mitochondrial ROS signaling in malignant lymphocytes and responses can vary between tumor types (59).

ROS formation and PHD activity can be ruled out as modulators for mitochondrial dependent HIF stabilization as shown here and by others (22, 50, 55). Similar observations have been made previously in severe mtDNA modulated cell lines, as well as the non-involvement of total glutathione levels (19), which also has been observed in fibroblasts from which our cybrids have been generated (17). In contrast, others indicate that mitochondrial ROS production is necessary for HIF-1α stabilization (60), even when a functional OXPHOS is not present (61). In addition to its role in proliferation, survival, angiogenesis and metastasis, HIF-1 is also involved in the regulation of tumor cell metabolism (2, 62, 63).

Our findings show that the time for tumor take was longer for mutant hybrid xenografts, but growth rates were not altered once the tumor was established when compared to control tumors. Previously, it has been shown that HIF-1α is responsible for tumor establishment, since HIF-1α knockdown resulted in a longer time needed to reach 60 mm^3^, while no differences in tumor growth rate was observed once tumors were established (64). In agreement, HIF-1α expression levels were reduced for the mutant hybrid xenografts. Furthermore, most of the tumors established from the mutant cell line lost their m.3243A>G mutation *in vivo*, possibly explaining the tumor take as we observed. Importantly, repopulation events in tumors harboring a mtDNA mutation or depletion should be taken into consideration, which eventually can lead to activation of the HIF-1 pathway and resulting tumor growth (65), a phenomenon also observed in our xenograft model. The presence of HIF-1α in cell lines with lower mutation percentage supports this observation. Another possible explanation of the absence of HIF-1α stabilization might be the involvement of the AMPK-mTOR pathway, which regulates energetic stress responses under normoxic conditions, as for instance the CI inhibitor metformin used in our experiments causes inhibition of mTOR (66, 67). However, additional experiments are necessary to evaluate if OXPHOS inhibition is inducing alterations in AMPK-mTOR signaling resulting in the observed HIF-1α deregulation by altered mRNA translation. Alterations in general mRNA translation are influenced upstream by the unfolded protein response (UPR), a mechanism influenced by hypoxia and energetic stress (32–34), but also these relationships need further investigations.

## Conclusions

This study explored the influence of mitochondrial function in the regulation of CAIX expression. The induction of mitochondrial dysfunction, by either depletion or by point mutations causing a severe mitochondrial phenotype, or inhibition of CI leads to a decreased CAIX expression. In our study, we observed in a MELAS cybrid model (m.3243A>G encoding for MT-TL1) that indeed CAIX mRNA and protein expression was reduced under hypoxic conditions. These findings suggest a general mitochondrial mechanism independent of its origin (either mtDNA of nDNA) of the mitochondrial gene (19, 50). Although the data on CI pharmacological inhibition were not always as clear as for the genetic cellular models, the differences in effects could probably be explained by cell type dependent differences. Here, similar results were observed for another HIF-1α target, VEGF caused by a reduced HIF-1α stabilization. Potentially this reduced HIF-1α stabilization phenotype can lead to a reduced tumor take *in vivo* however the precise underlying mechanisms should be further elucidated.

## Data Availability Statement

All datasets generated for this study are included in the article/Supplementary Material.

## Ethics Statement

The animal studies were reviewed and approved by the Animal Ethics Committee of the University Maastricht (ethic code: 2014–115).

## Author Contributions

MG and LD designed the experiments. MG preformed the main experiments while KO, AV, NL, RB, and RH performed additional experiments. MG, AV, and LD analyzed the data. MG, AV, LD, and PL wrote, reviewed, and edited the manuscript.

## Conflict of Interest

AV and PL report grants from Netherlands Genomics Initiative (NGI), during the conduct of the study; In addition, AV and PL have a patent US2016160287 (entitled “Method for determining the risk of developing radiation-induced toxicity after exposure to radiation”) licensed to ptTheragnostics. The remaining authors declare that the research was conducted in the absence of any commercial or financial relationships that could be construed as a potential conflict of interest.

## Supplementary Material

Supplementary Figure S1 Experimental data of A549 cells **(A)** Merged images of dsDNA staining (green) and mitochondria (red). **(B)** Percentage mtDNA copy number. **(C)** CAIX mRNA expression upon hypoxia. **(D)** Representative Western blot of CAIX protein expression upon normoxia (–) or hypoxia (+). **(E)** CAIX mRNA expression upon hypoxia with or without Metformin (5 mM) or Rotenone (1 μM). **(F)** Representative Western blot and quantification of CAIX protein expression upon normoxia (–) or hypoxia (+) with or without Metformin (M, 5 mM) or Rotenone (R, 1 μM). **(G)** VEGF and **(H)** HIF-1α mRNA expression under hypoxia. **(I)** Representative Western blot and quantification of HIF-1α protein expression upon normoxia (–) or hypoxia (+), combined with exposure of vehicle (–), metformin (M) or rotenone (R). Data represent the mean + SEM of 2≥ biological repeats. ^*^*p* < 0.05 ^**^*p* < 0.01.

Supplementary Figure S2 CAIX mRNA expression. CAIX mRNA expression levels for 143B parental and 143B ρ^0^ cells upon normoxia and hypoxia. Data are normalized to untreated (–) normoxia levels for 143B parental cells. R = rotenone, M = metformin. *N* ≥ 2; Mean + SEM.

Supplementary Figure S3 Metabolic profiling of the cybrid lines. **Top panel**: Oxygen consumption rate measured before and after the injection of oligomycin (1 μM); FCCP (0.5 μM) and a combination of Rotenone (1 μM) and Antimycin A (1 μM). **Bottom panel**: ATP production, proton leakage and spare capacity calculations of the trace in panel 1 of control 1, control 2 and MELAS 2 cell lines. *N* ≥ 3; Mean ± SEM.

Supplementary Figure S4 Representative blot of CAIX expression for control and MELAS cybrid lines upon exposure to hypoxia.

Supplementary Figure S5 CAXII mRNA expression. **Left panel**: CAXII mRNA expression levels for 143B parental and 143B ρ0 cells upon hypoxia. **Right panel**: CAXII mRNA expression for levels for cybrid (m.3243 A>G mutant) cells and control cells upon hypoxia. Data are normalized to either parental or control cells. *N* = 2; Mean + SEM.

Supplementary Figure S6 ROS production. **Top panel**: ROS levels for 143B parental and 143B ρ^0^ cells upon normoxia and hypoxia. **Bottom panel**: ROS levels for cybrid (m.3243 A>G mutant) cells and control cells upon normoxia and hypoxia. Data are normalized to untreated (–) normoxia levels of either parental or control 1 cells. R = rotenone, M = metformin. *N* = 3; Mean + SEM.

Supplementary Figure S7 HIF-2 protein expression **Left panel**: Representative Western blot of HIF-2 expression in xenografts harboring a m.3243A>G mutation **Right panel**: quantification of HIF-2 protein expression normalized to actin levels.

Supplementary Figure S8 SIRT3 mRNA expression upon normoxia (gray) and hypoxia (white) (top panel) and the ratio between normoxia and hypoxia (bottom panal). Data represent the mean + SEM of three biological repeats. ^*^*p* < 0.05.

Supplementary Table 1 Primer sequences for quantitative real-time PCR and fragment analysis.
