# Supplementary figures and images for: Mitochondrial Dysfunction Inhibits Hypoxia-Induced HIF-1α Stabilization and Expression of Its Downstream Targets
Source: Front Oncol. 2020 May 19;10:770. doi: 10.3389/fonc.2020.00770 (PMC7248342; doi:10.3389/fonc.2020.00770)

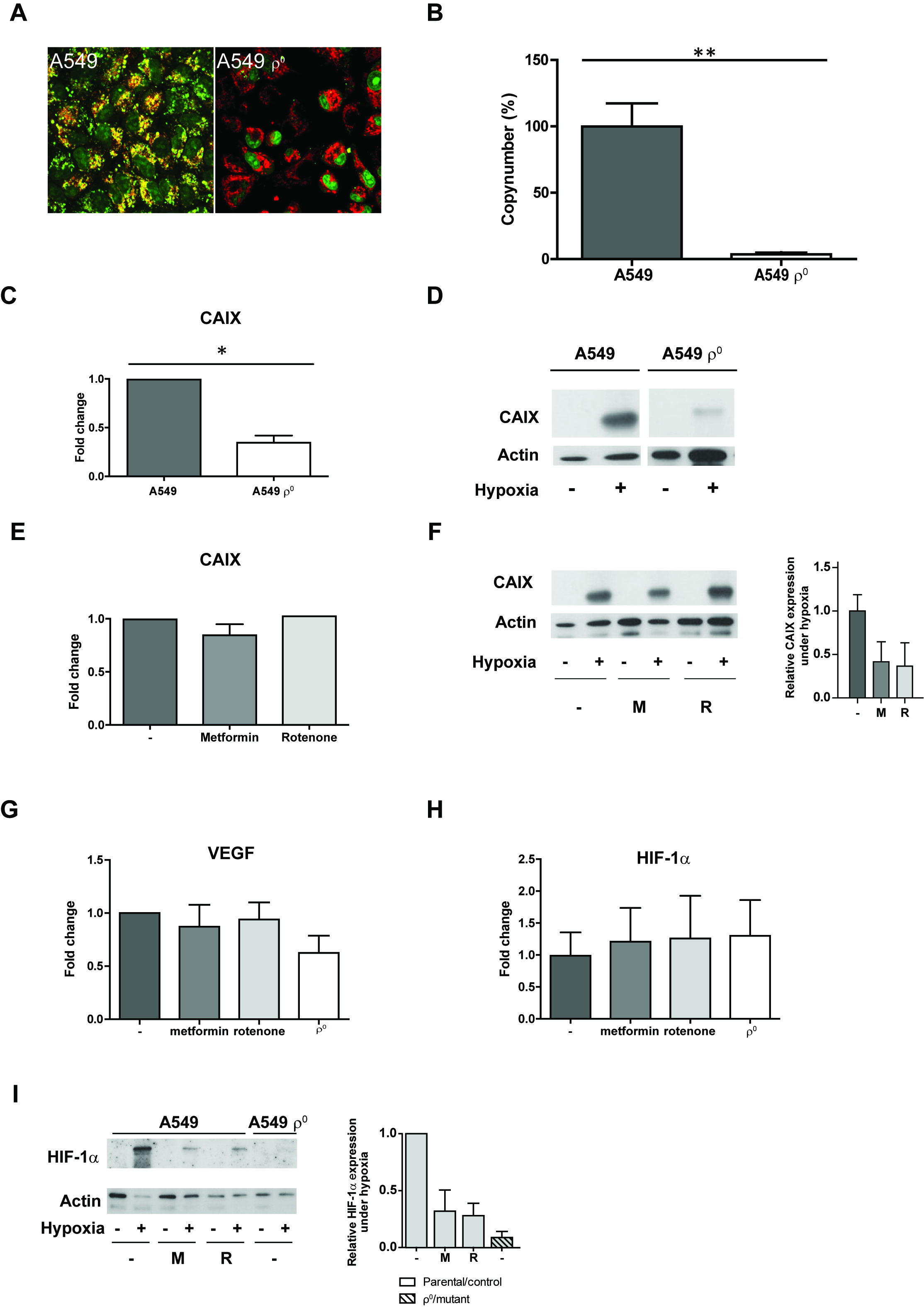

Supplement: Supplementary Figure S1 — Experimental data of A549 cells (A) Merged images of dsDNA staining (green) and mitochondria (red). (B) Percentage mtDNA copy number. (C) CAIX mRNA expression upon hypoxia. (D) Representative Western blot of CAIX protein expression upon normoxia (–) or hypoxia (+). (E) CAIX mRNA expression upon hypoxia with or without Metformin (5 mM) or Rotenone (1 μM). (F) Representative Western blot and quantification of CAIX protein expression upon normoxia (–) or hypoxia (+) with or without Metformin (M, 5 mM) or Rotenone (R, 1 μM). (G) VEGF and (H) HIF-1α mRNA expression under hypoxia. (I) Representative Western blot and quantification of HIF-1α protein expression upon normoxia (–) or hypoxia (+), combined with exposure of vehicle (–), metformin (M) or rotenone (R). Data represent the mean + SEM of 2≥ biological repeats. *p < 0.05 **p < 0.01. [file Image_1.TIF]

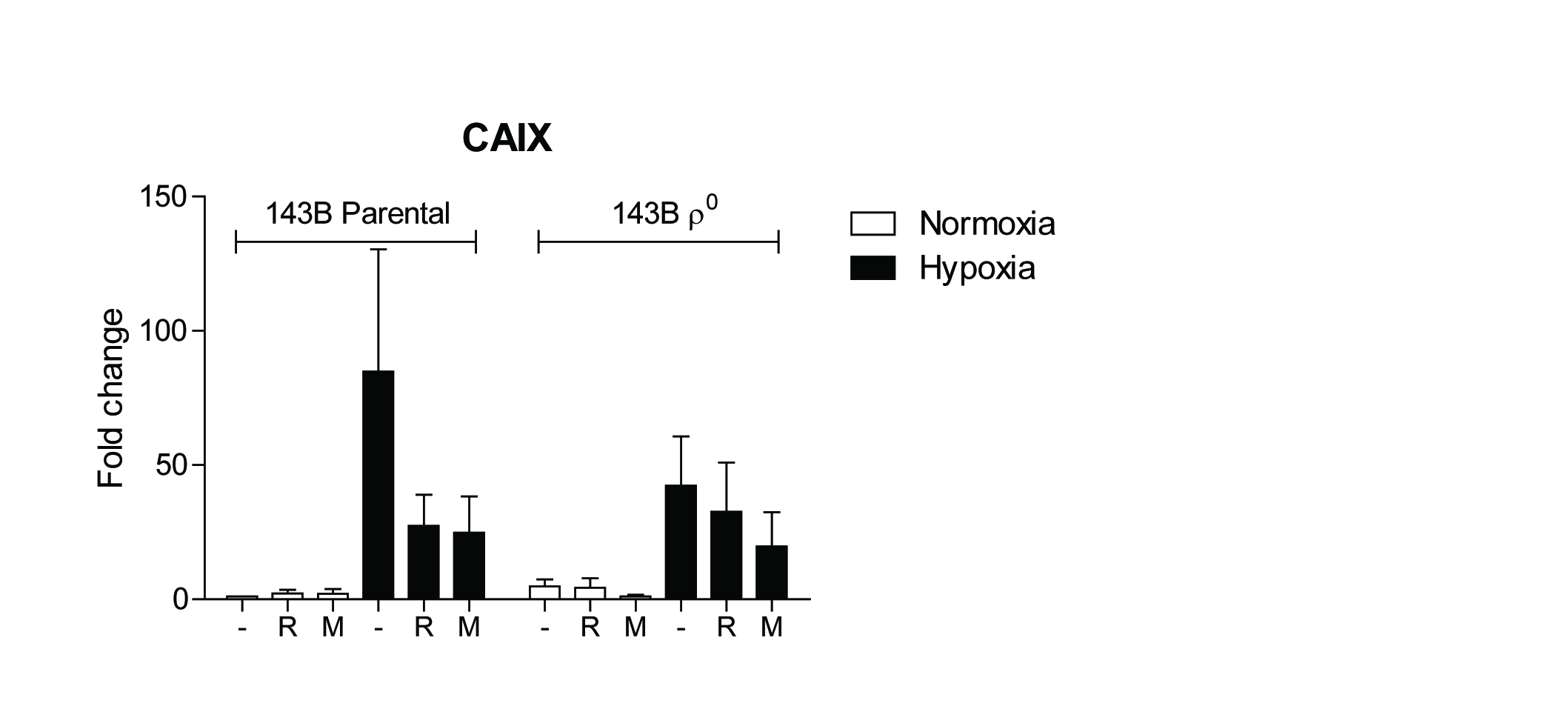

Supplement: Supplementary Figure S2 — CAIX mRNA expression. CAIX mRNA expression levels for 143B parental and 143B ρ0 cells upon normoxia and hypoxia. Data are normalized to untreated (–) normoxia levels for 143B parental cells. R = rotenone, M = metformin. N ≥ 2; Mean + SEM. [file Image_2.TIF]

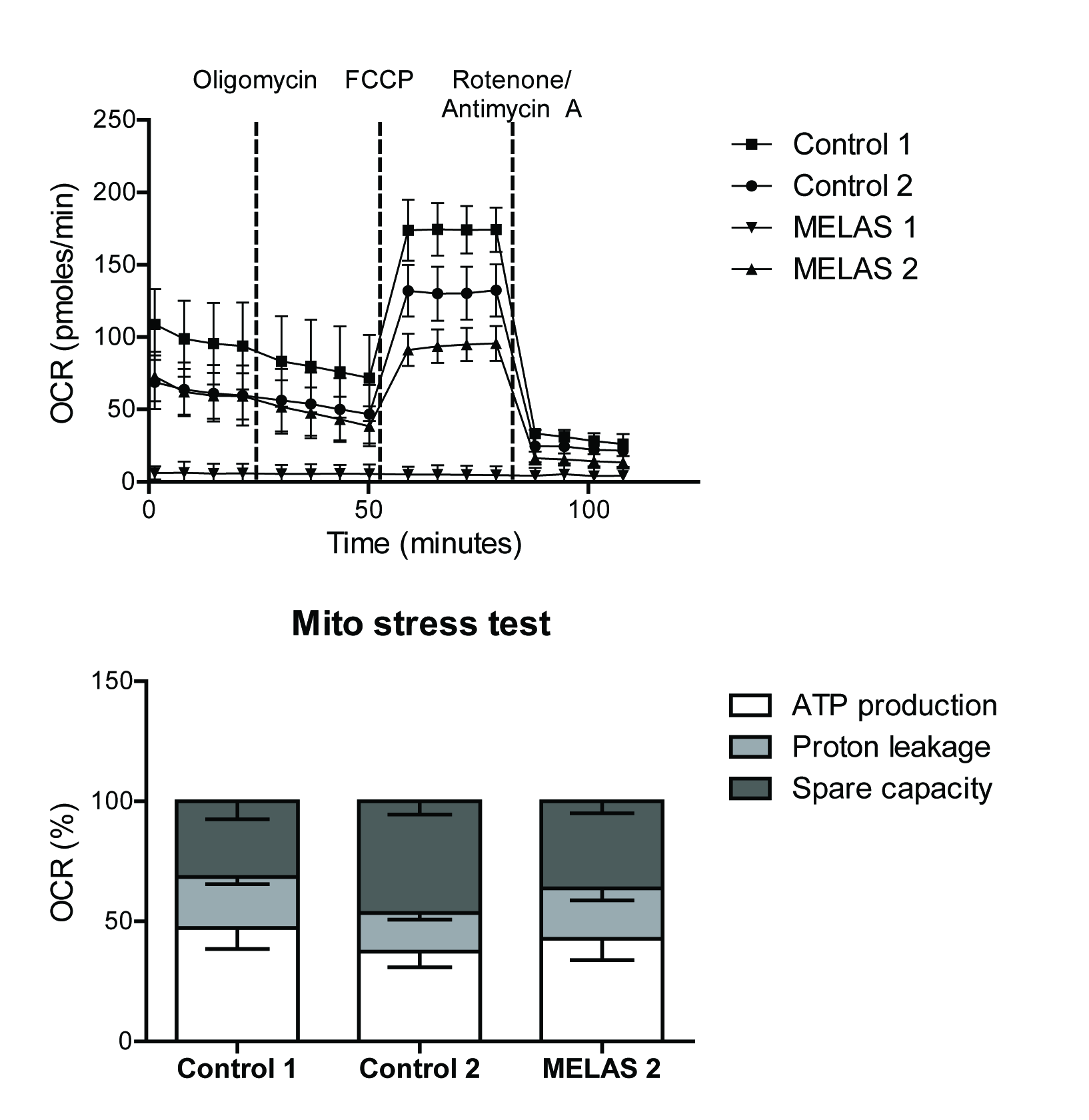

Supplement: Supplementary Figure S3 — Metabolic profiling of the cybrid lines. Top panel: Oxygen consumption rate measured before and after the injection of oligomycin (1 μM); FCCP (0.5 μM) and a combination of Rotenone (1 μM) and Antimycin A (1 μM). Bottom panel: ATP production, proton leakage and spare capacity calculations of the trace in panel 1 of control 1, control 2 and MELAS 2 cell lines. N ≥ 3; Mean ± SEM. [file Image_3.TIF]

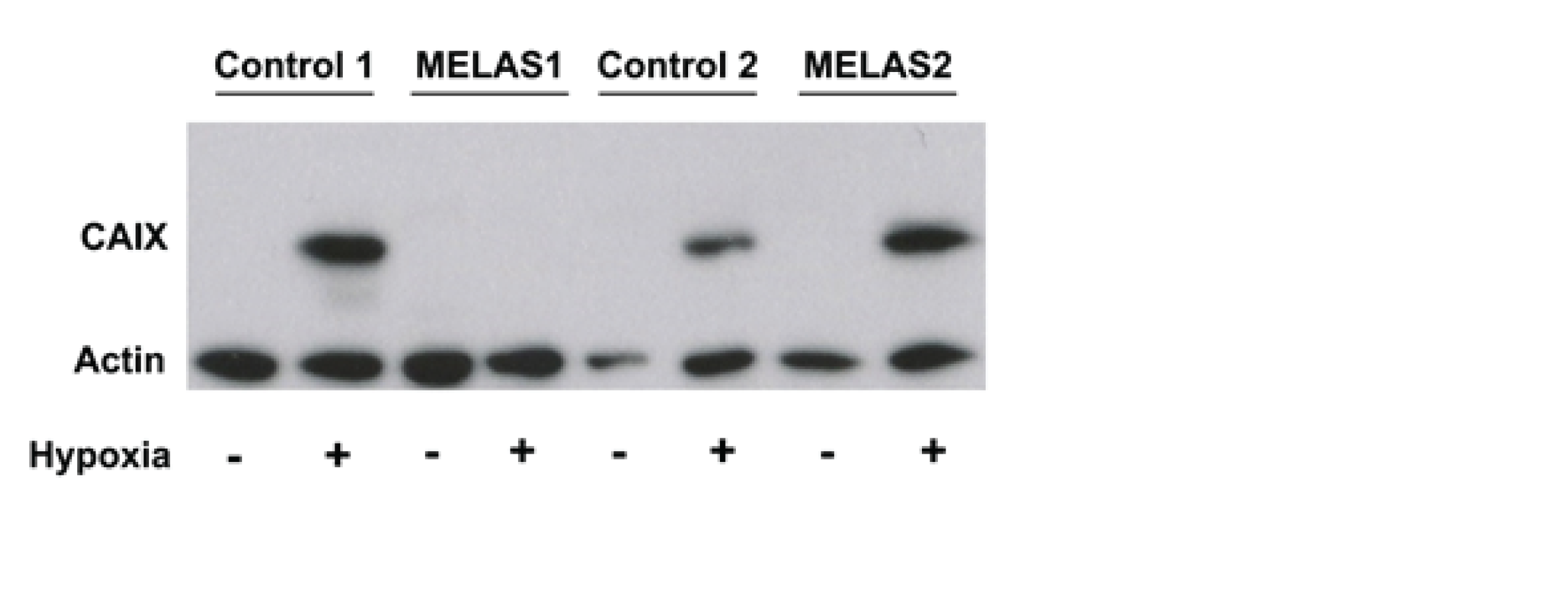

Supplement: Supplementary Figure S4 — Representative blot of CAIX expression for control and MELAS cybrid lines upon exposure to hypoxia. [file Image_4.TIF]

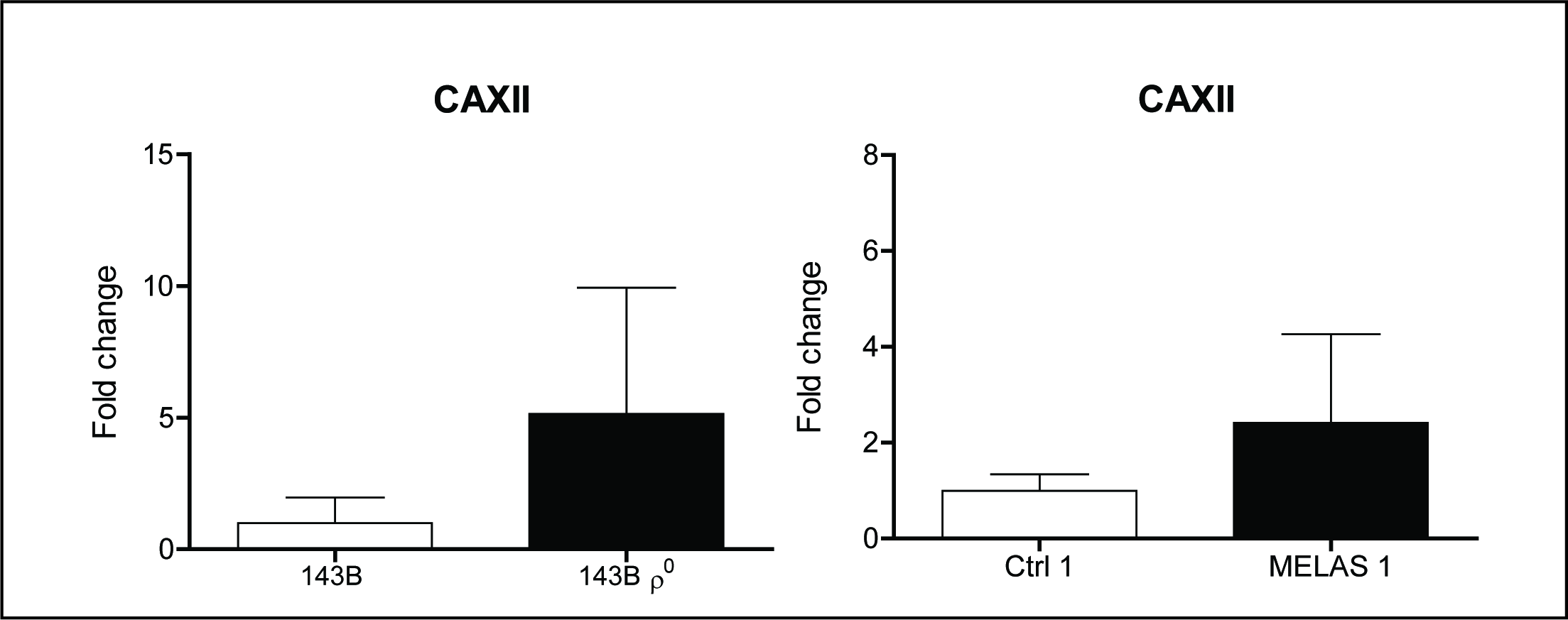

Supplement: Supplementary Figure S5 — CAXII mRNA expression. Left panel: CAXII mRNA expression levels for 143B parental and 143B ρ0 cells upon hypoxia. Right panel: CAXII mRNA expression for levels for cybrid (m.3243 A>G mutant) cells and control cells upon hypoxia. Data are normalized to either parental or control cells. N = 2; Mean + SEM. [file Image_5.TIF]

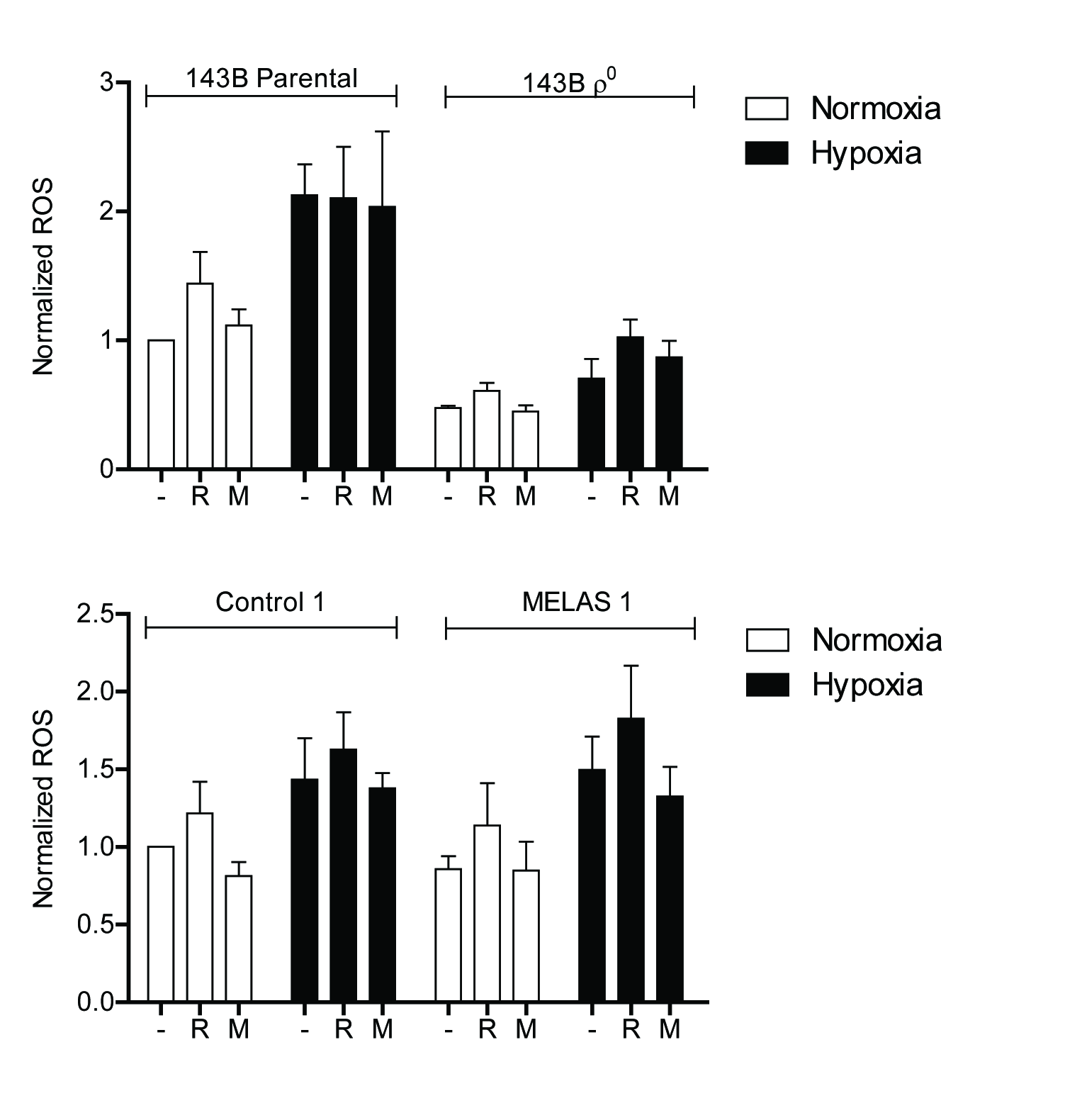

Supplement: Supplementary Figure S6 — ROS production. Top panel: ROS levels for 143B parental and 143B ρ0 cells upon normoxia and hypoxia. Bottom panel: ROS levels for cybrid (m.3243 A>G mutant) cells and control cells upon normoxia and hypoxia. Data are normalized to untreated (–) normoxia levels of either parental or control 1 cells. R = rotenone, M = metformin. N = 3; Mean + SEM. [file Image_6.TIF]

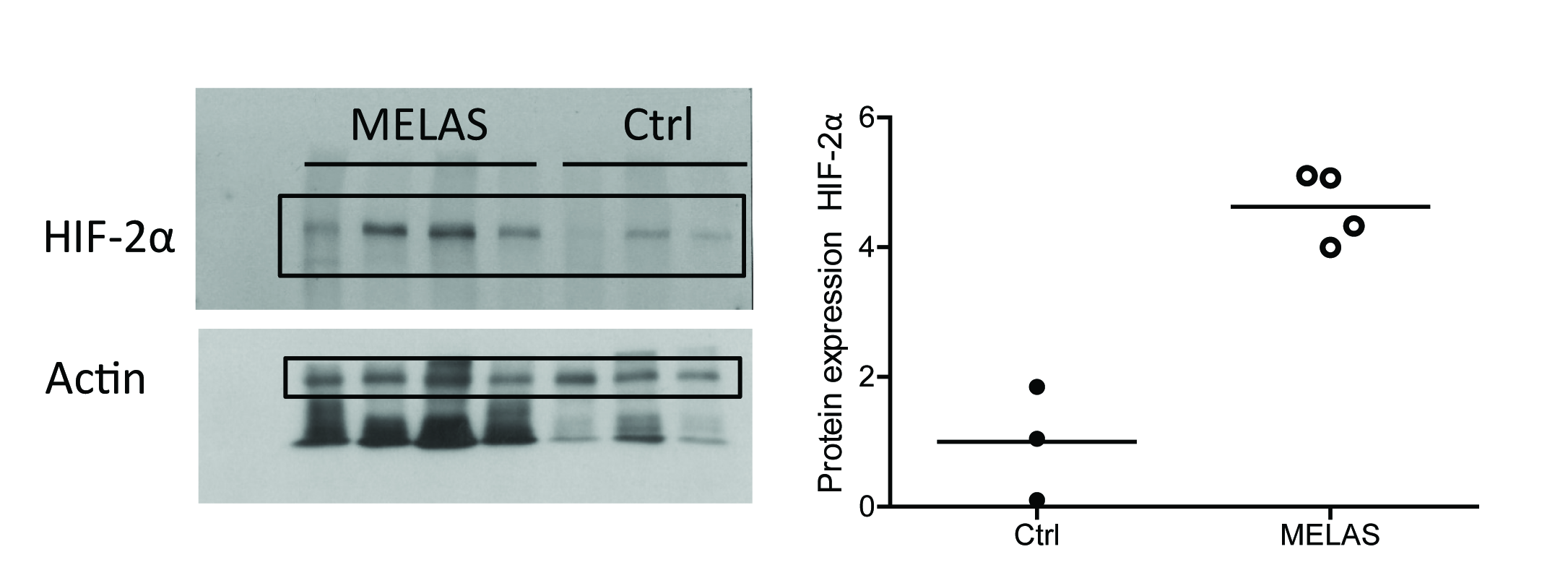

Supplement: Supplementary Figure S7 — HIF-2 protein expression Left panel: Representative Western blot of HIF-2 expression in xenografts harboring a m.3243A>G mutation Right panel: quantification of HIF-2 protein expression normalized to actin levels. [file Image_7.TIF]

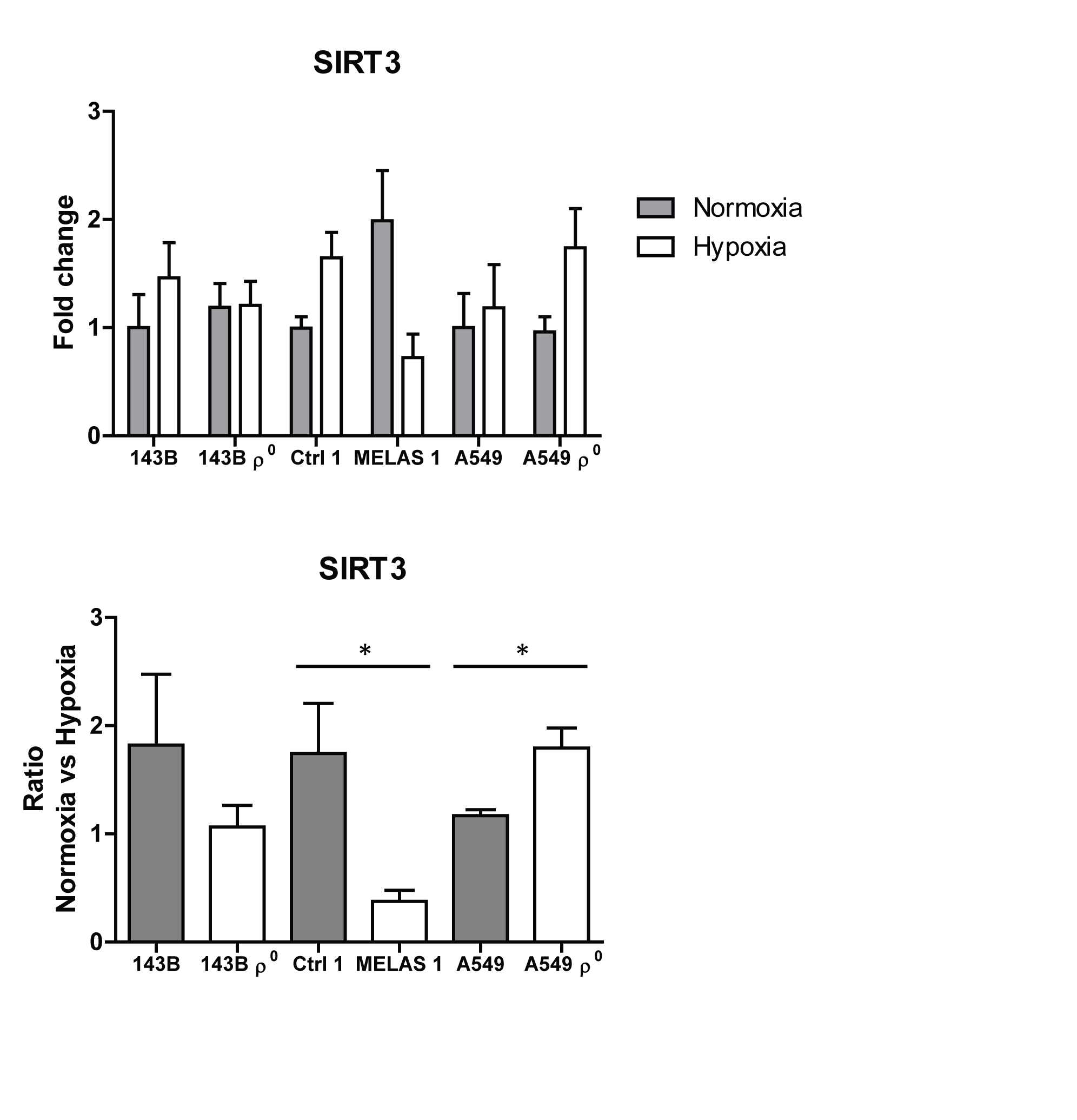

Supplement: Supplementary Figure S8 — SIRT3 mRNA expression upon normoxia (gray) and hypoxia (white) (top panel) and the ratio between normoxia and hypoxia (bottom panal). Data represent the mean + SEM of three biological repeats. *p < 0.05. [file Image_8.TIF]

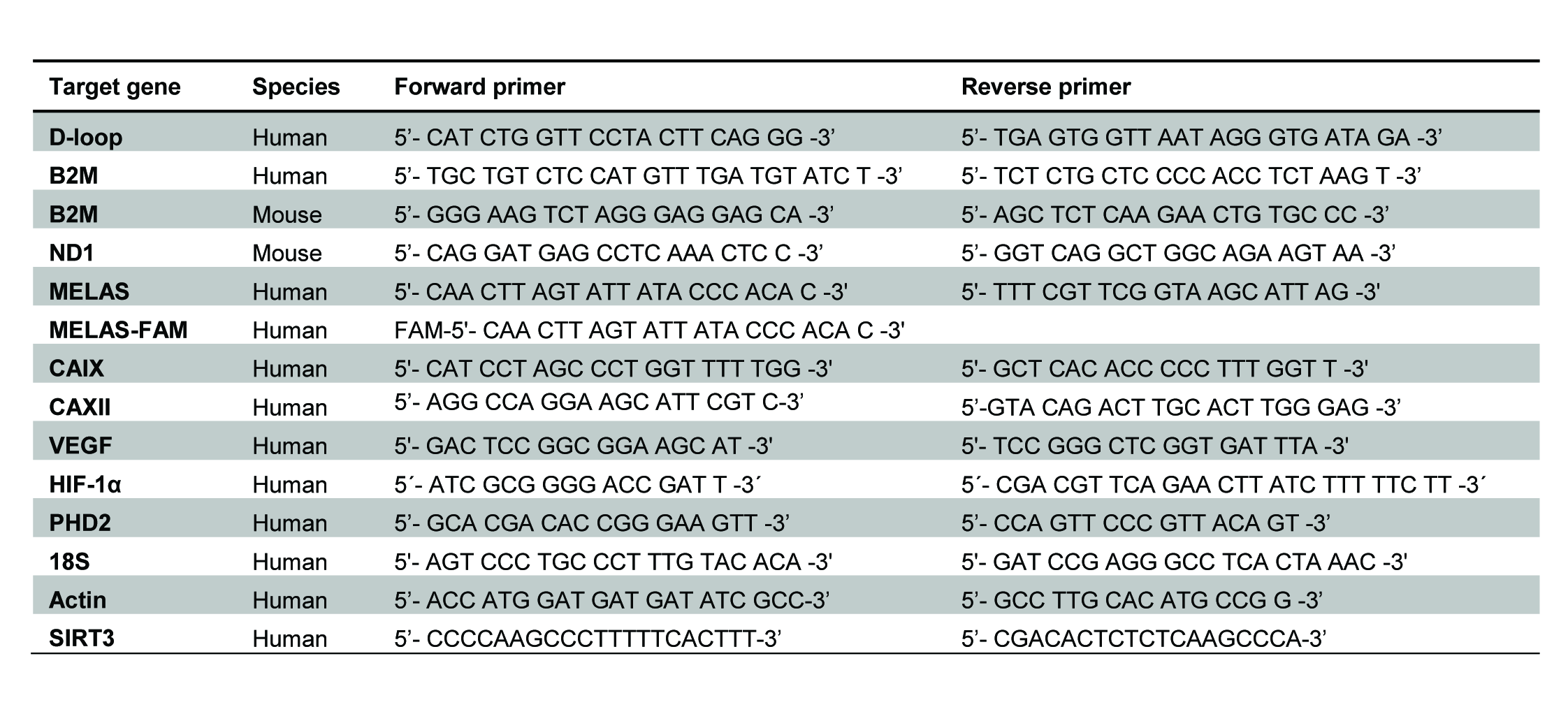

Supplement: Supplementary Table 1 — Primer sequences for quantitative real-time PCR and fragment analysis. [file Image_9.TIF]
