# Supplementary material for: Mitochondrial Dysfunction Inhibits Hypoxia-Induced HIF-1α Stabilization and Expression of Its Downstream Targets
Source: Front Oncol. 2020 May 19;10:770. doi: 10.3389/fonc.2020.00770 (PMC7248342; doi:10.3389/fonc.2020.00770)

Figure 1D complete blot

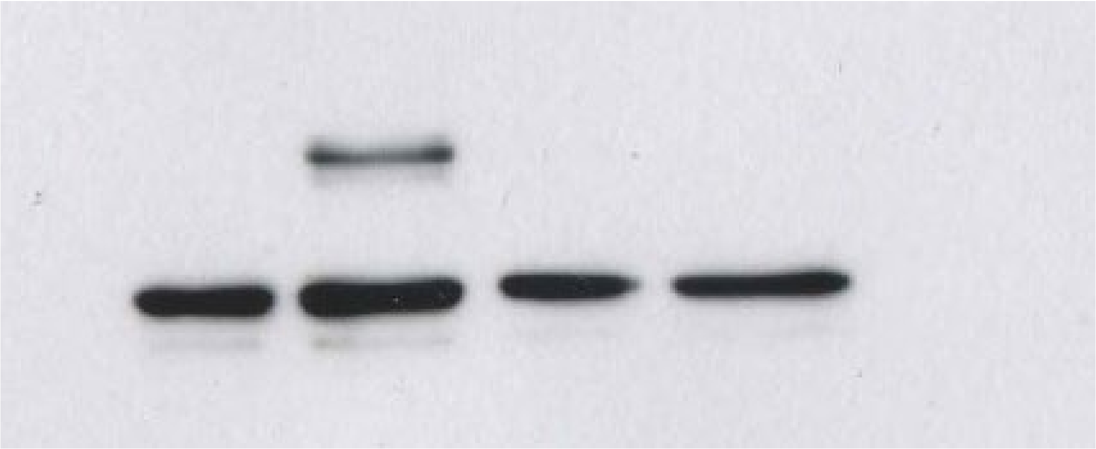

CAIX: 54 kD

Actin: 42 kD

Figure 2D complete blot

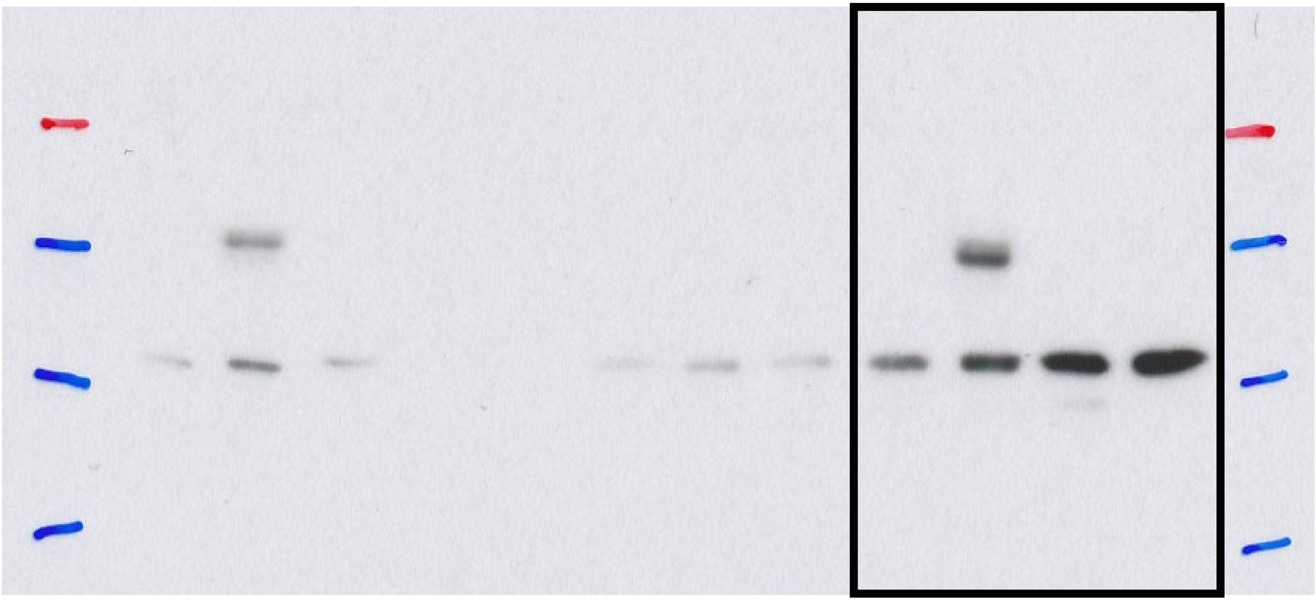

CAIX: 54 kD

Actin: 42 kD

Figure 3B 143B panel

CAIX: 54 kD

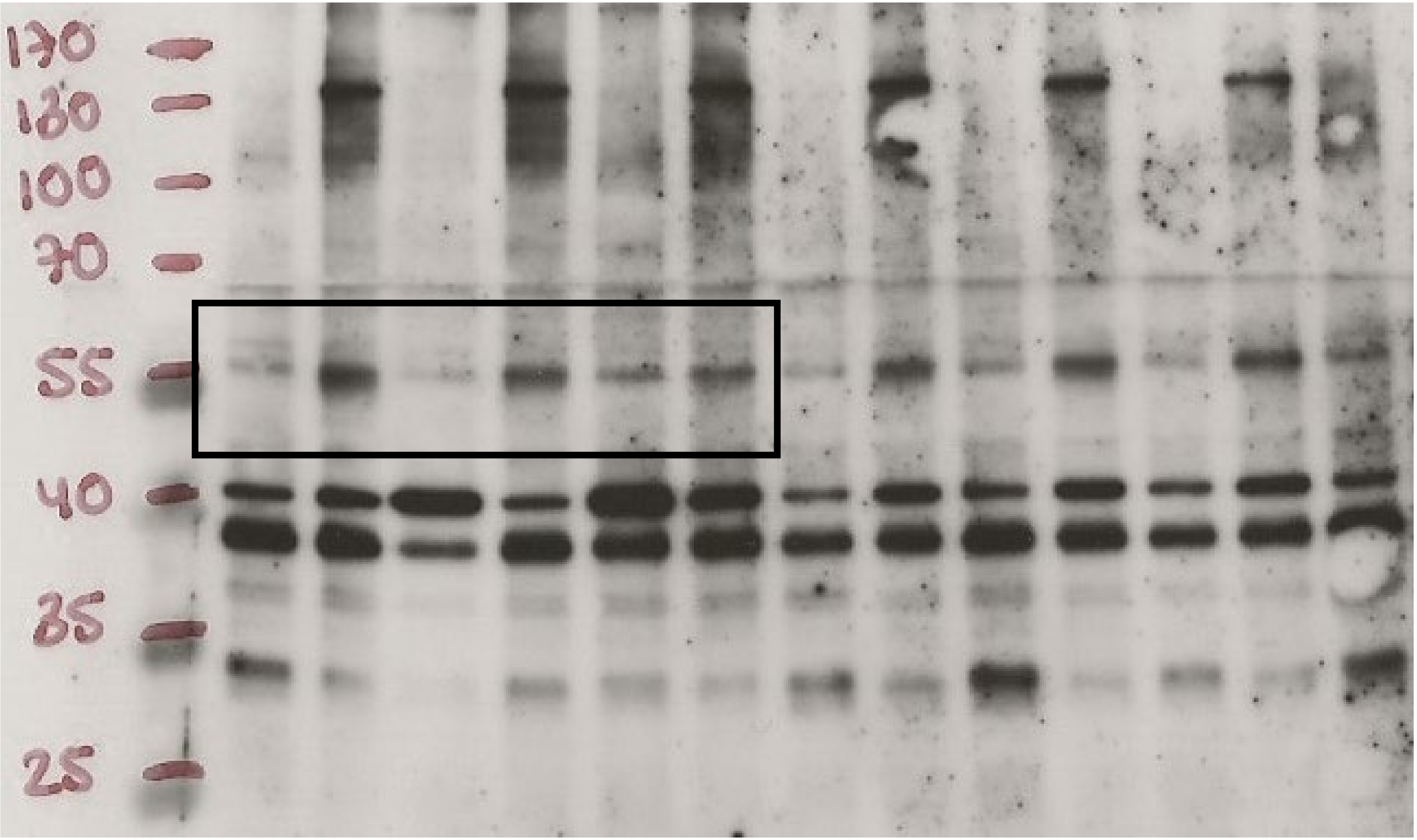

Figure 3B 143B panel

Shorter exposure

Actin: 42 kD

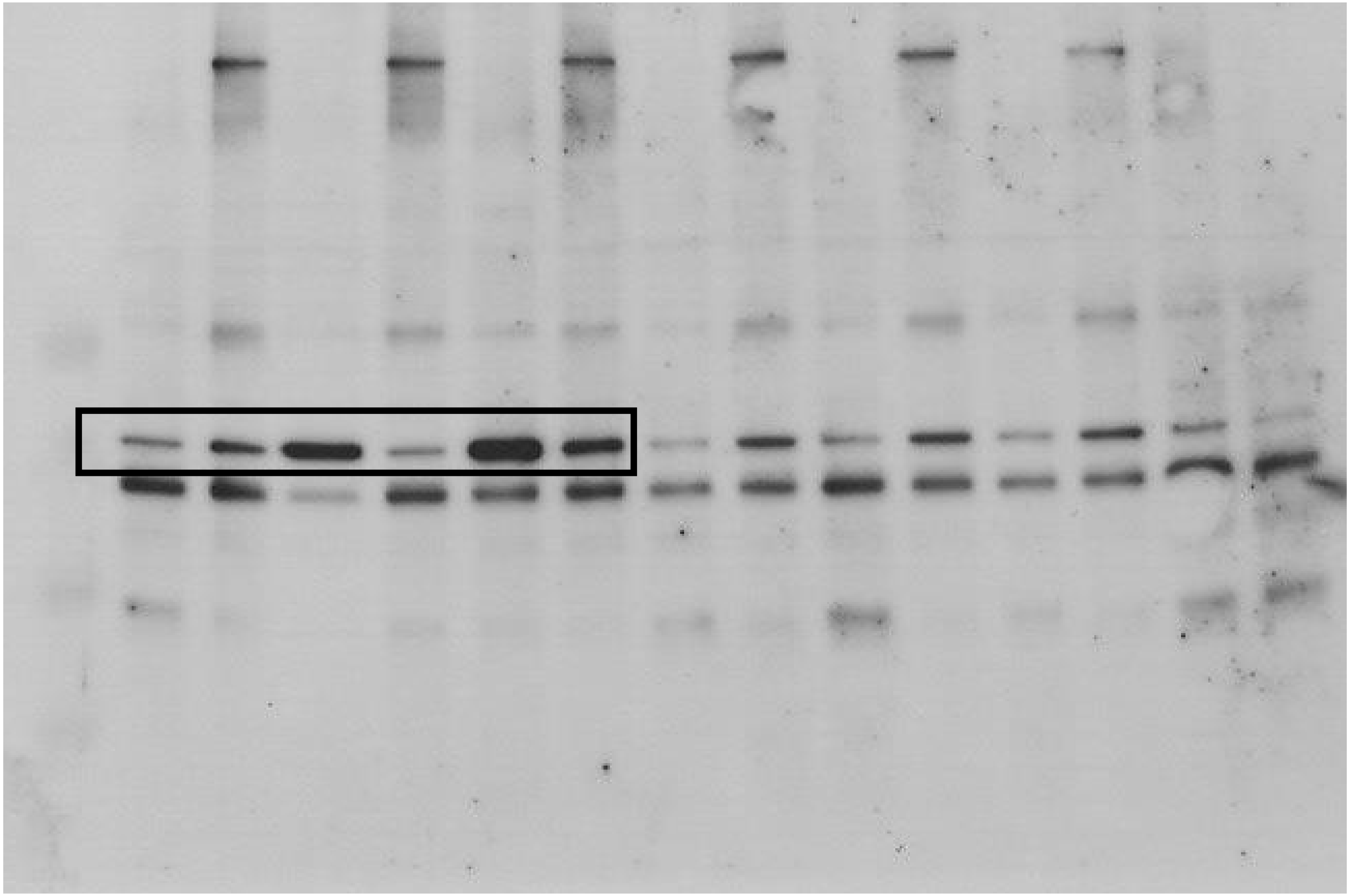

Figure 3B Control 1 panel

CAIX: 54 kD

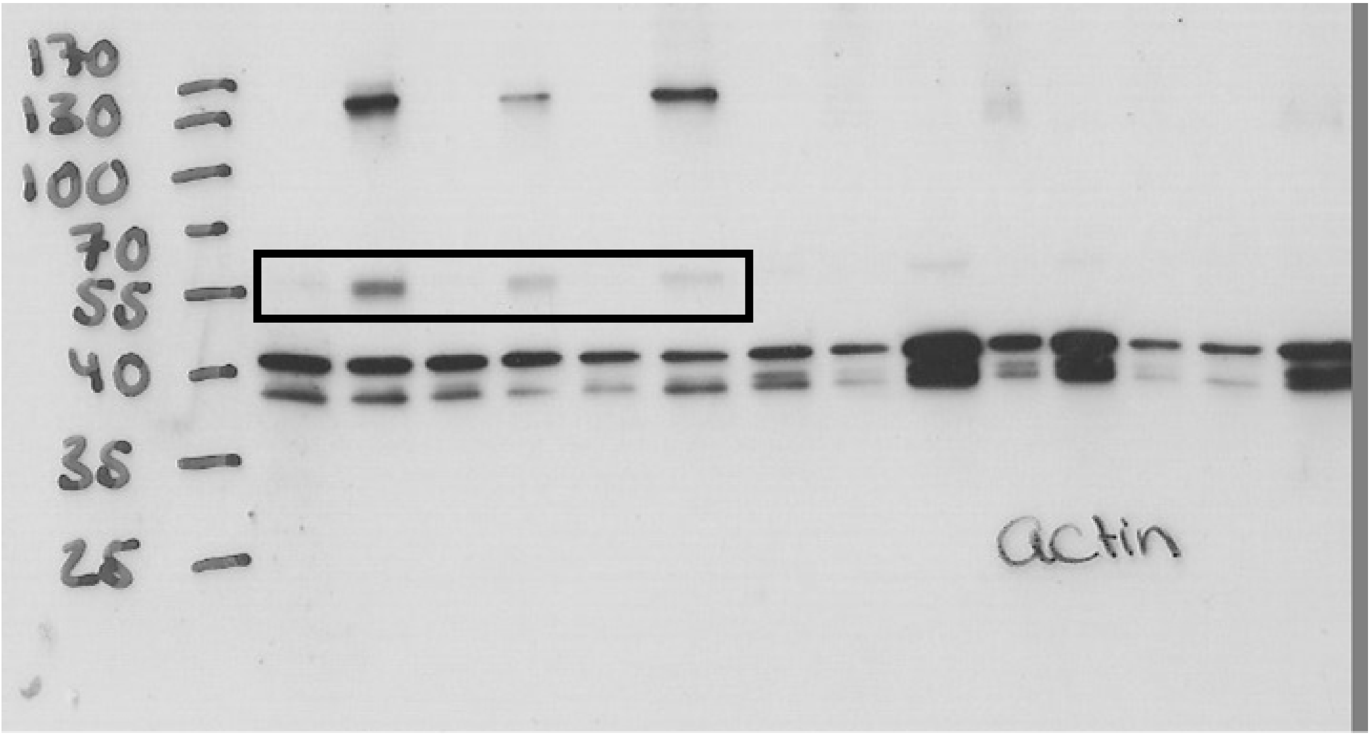

Figure 3B Control 1 panel

Shorter exposure

Actin: 42 kD

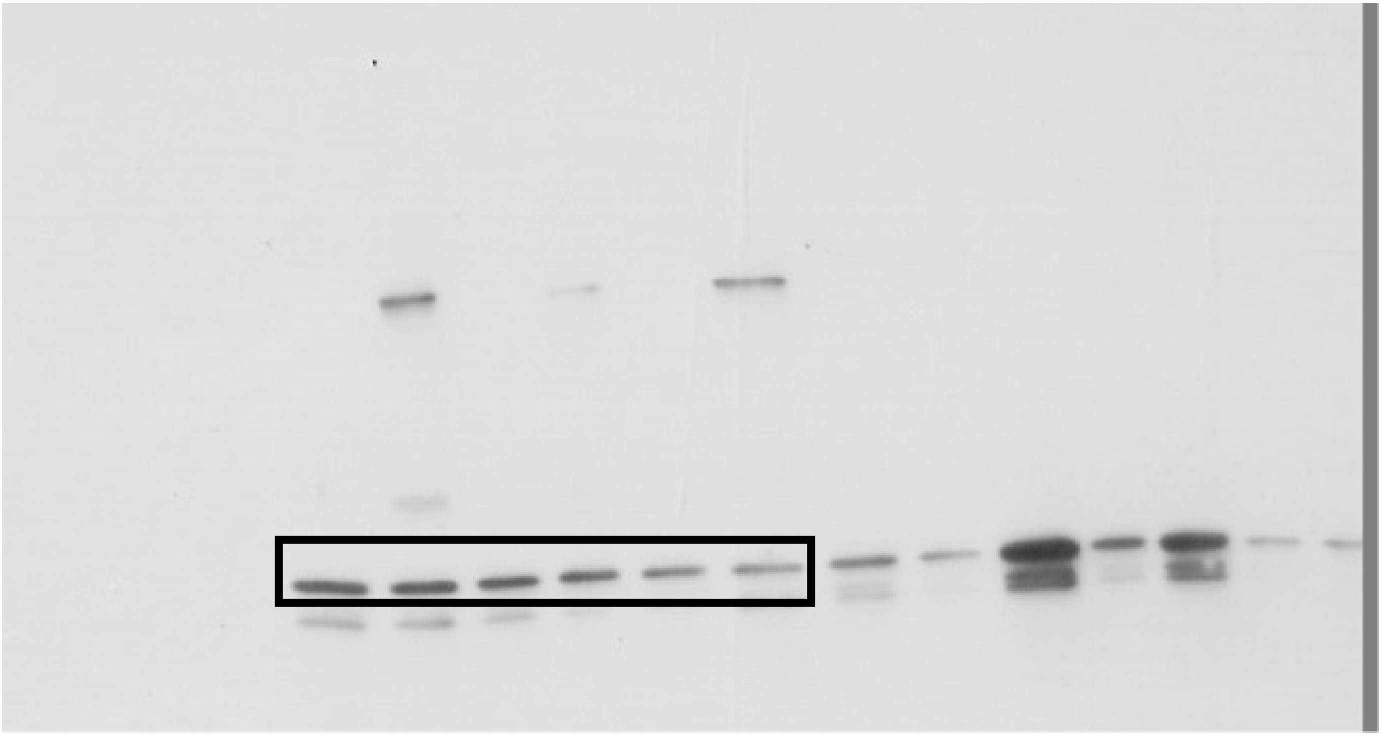

Figure 3E 143B panel

HIF1a: 120 kD

Actin: 42 kD

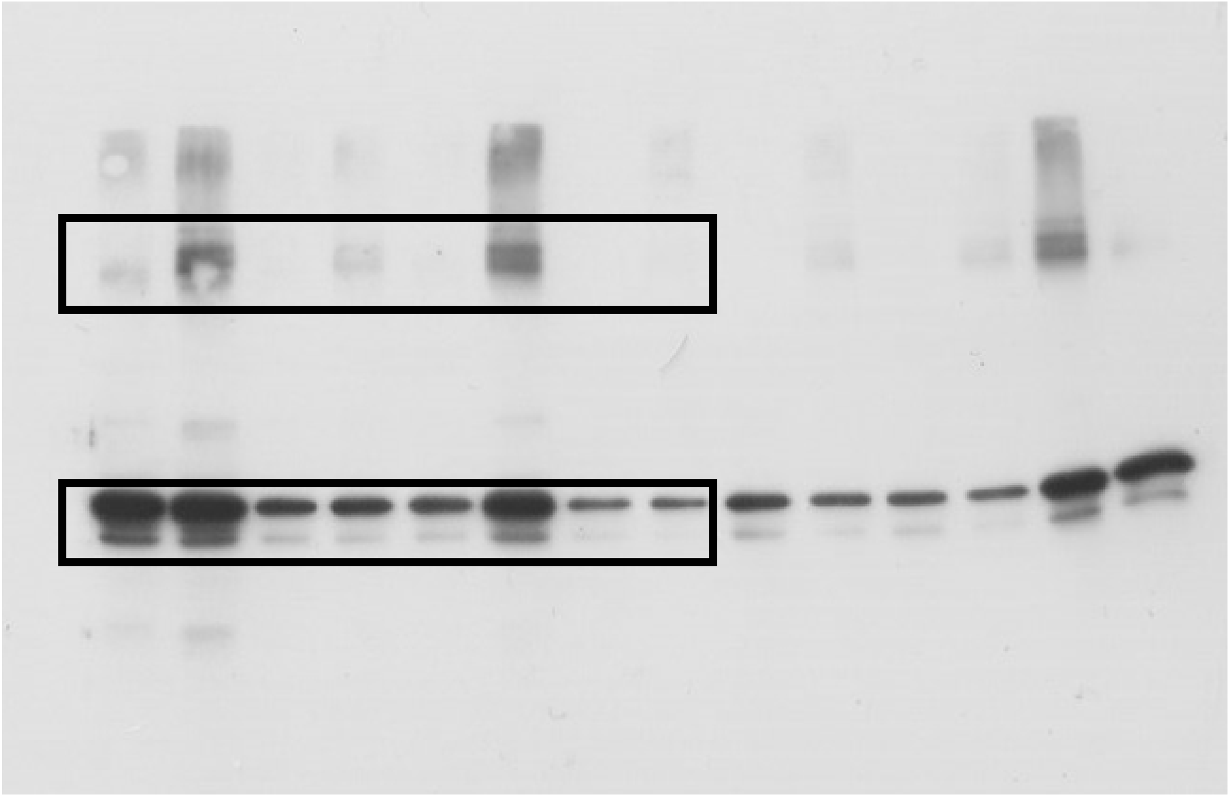

Figure 3E cybrid panel

HIF1a: 120 kD

Actin: 42 kD

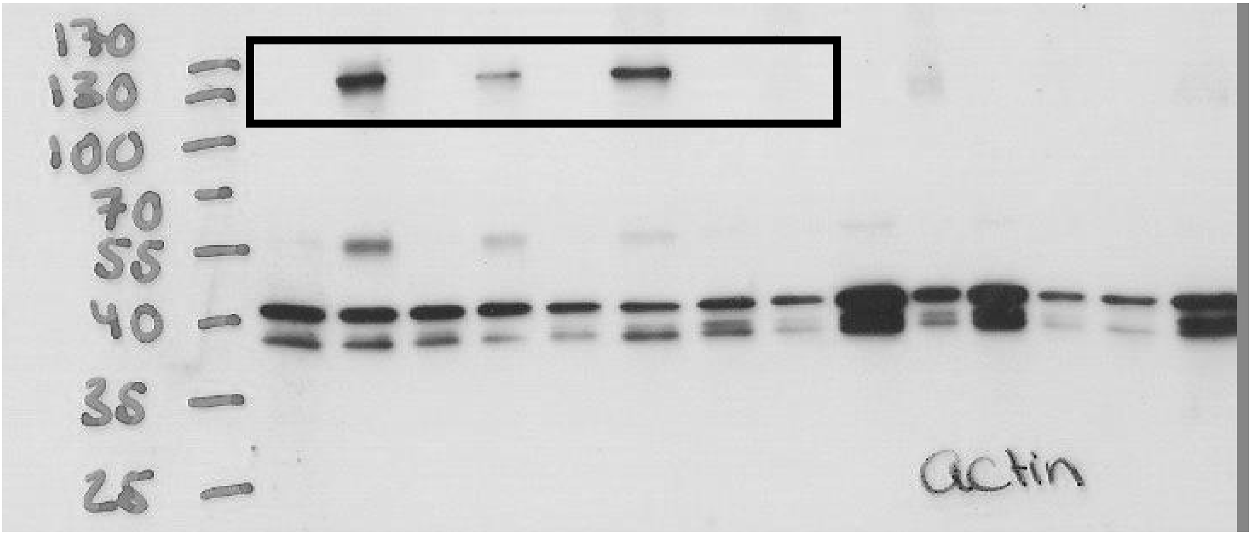

Figure 3E Cybrid panel

Shorter exposure

Actin: 42 kD

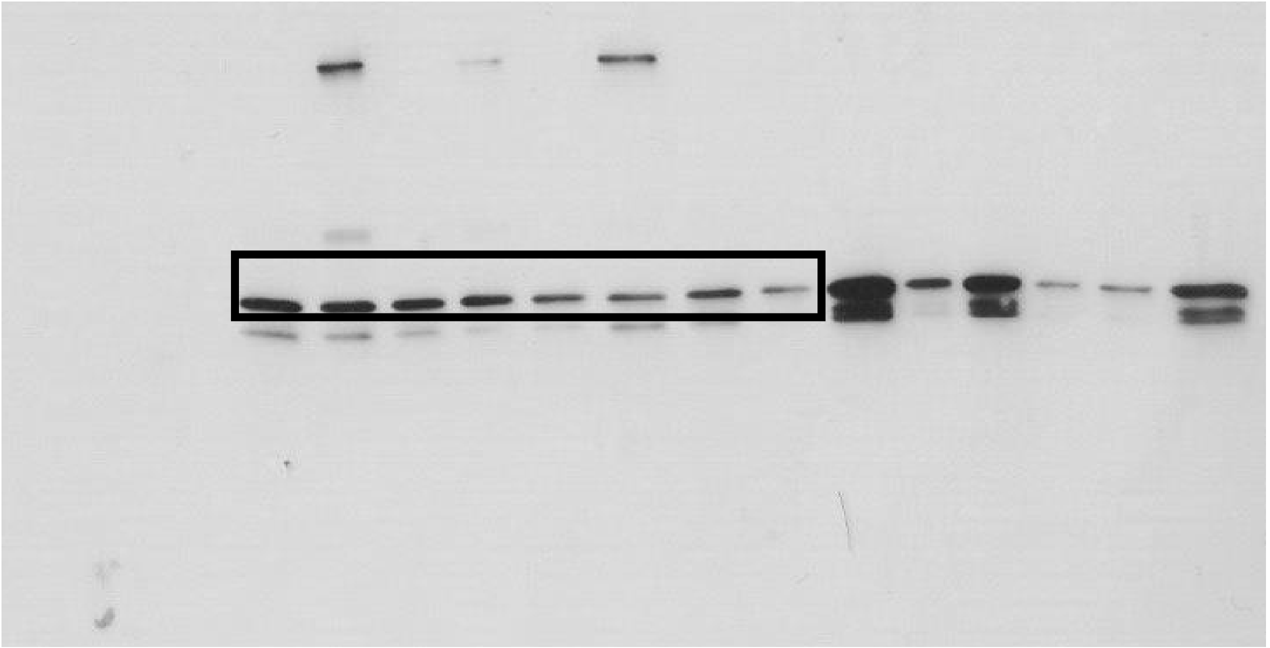

Figure 5C complete blot

HIF1a: 120 kD

Actin: 42 kD

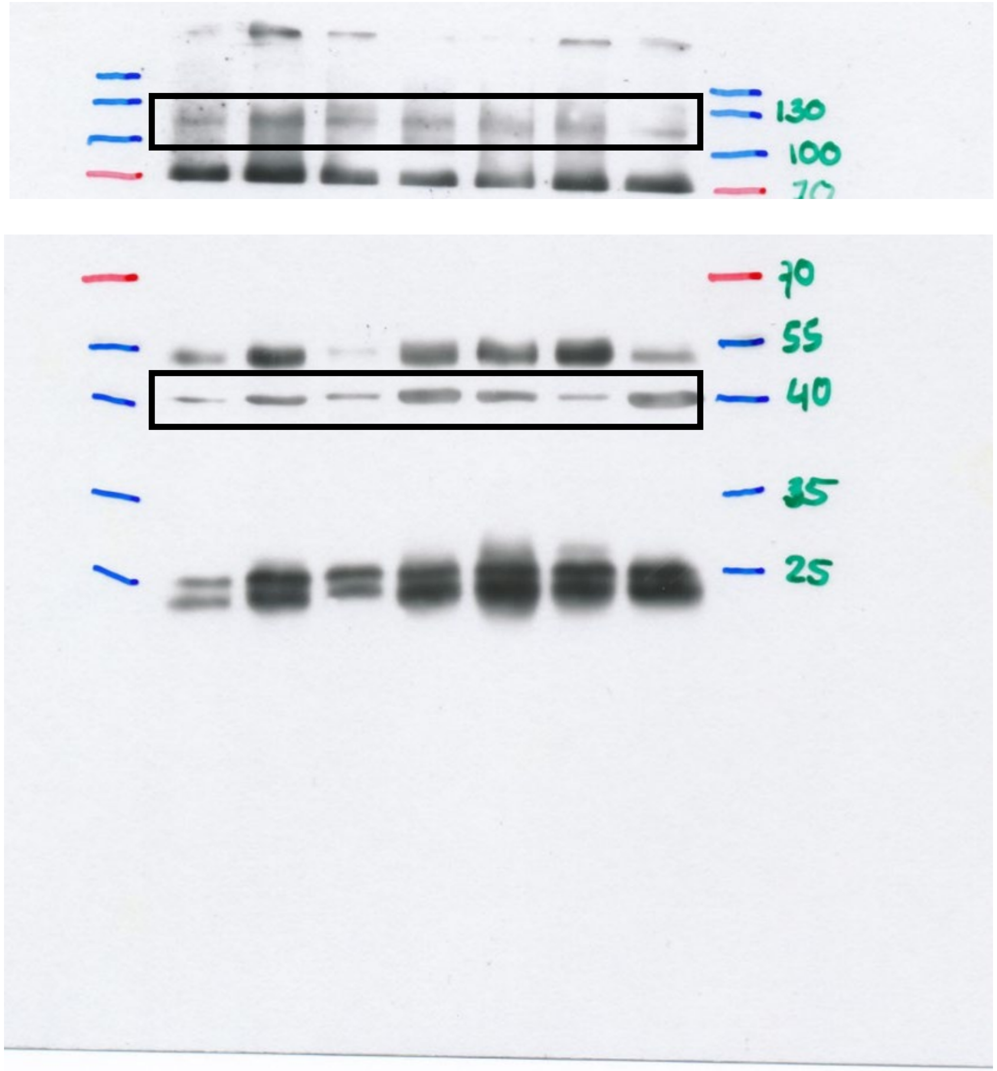

Supplementary Figure S1 D

CAIX: 54 kD

Actin: 42 kD

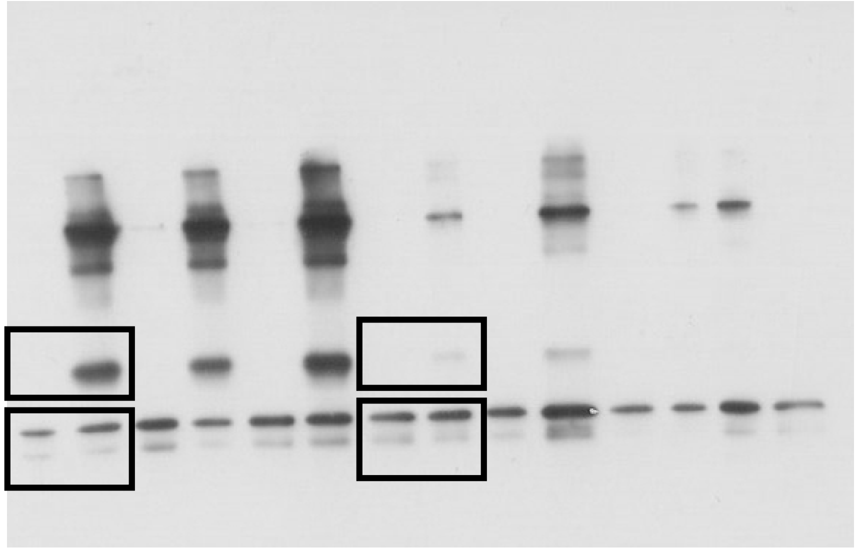

Supplementary Figure S1 F

HIF1a: 120 kD

Actin: 42 kD

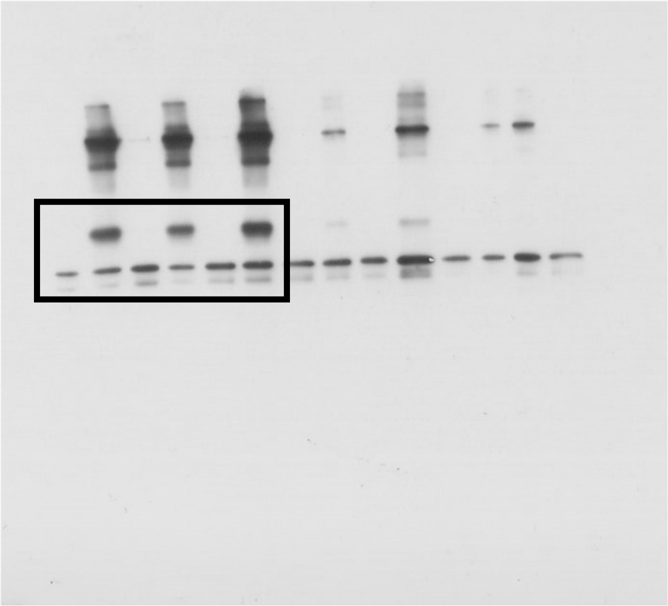

Supplementary Figure S1 I

HIF1a: 120 kD

Actin: 42 kD

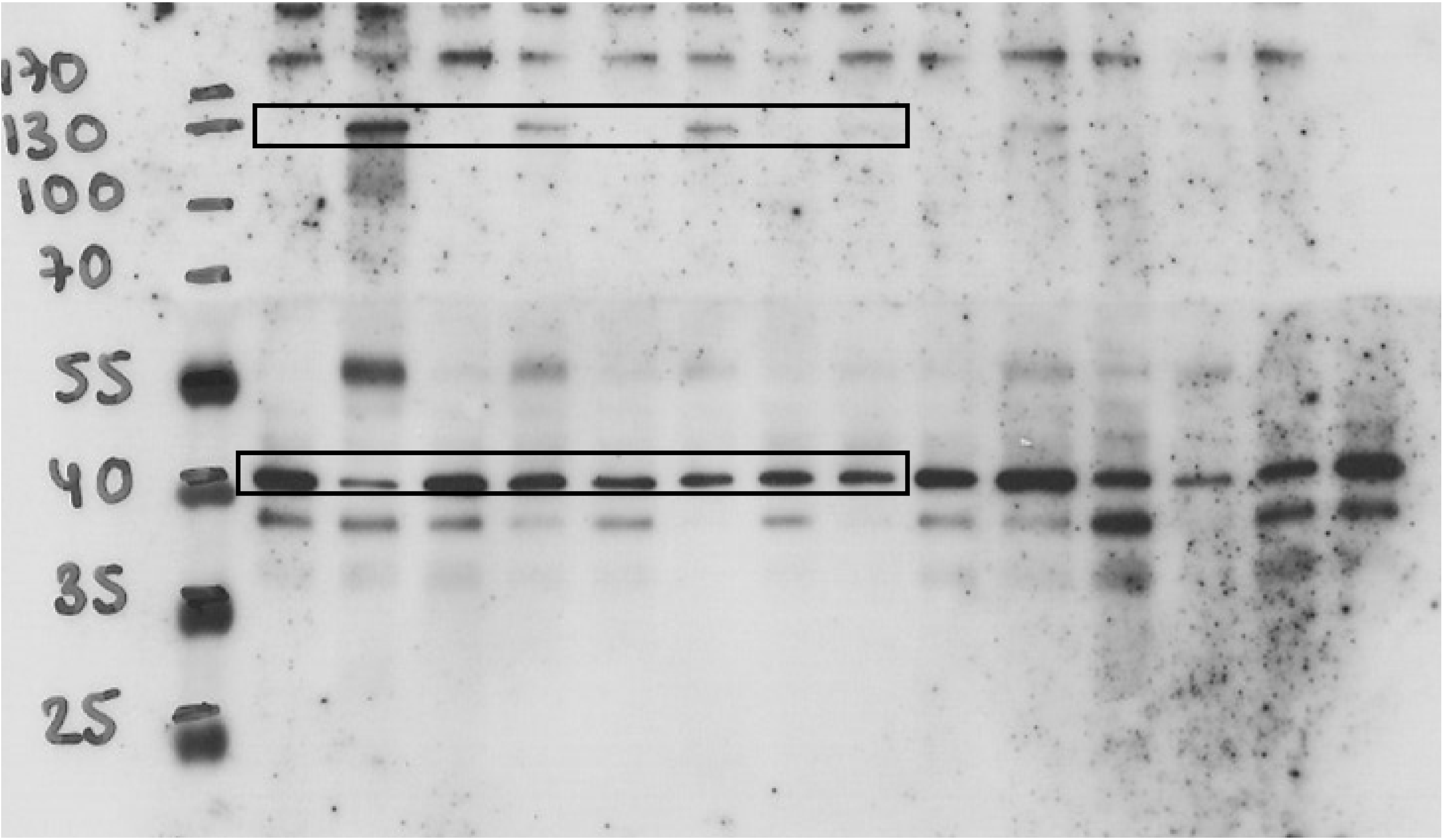

Supplement: Supplementary file 10 [file Data_Sheet_1.pdf]
